# Dynamics, phylogeny and phyto-stimulating potential of chitinase synthesizing bacterial root endosymbiosiome of North Western Himalayan *Brassica rapa* L.

**DOI:** 10.1038/s41598-022-11030-0

**Published:** 2022-04-25

**Authors:** Shahid Ahmad Padder, Rauoof Ahmad Rather, Sajad Ahmad Bhat, M. D. Shah, Tawseef Rehman Baba, N. M. Mubarak

**Affiliations:** 1 Division of Basic Sciences and Humanities, FoH, Sher-e-Kashmir University of Agricultural Sciences and Technology of Kashmir, Srinagar, Kashmir, J&K 190025 India; 2 Division of Environmental Sciences, FoH, Sher-e-Kashmir University of Agricultural Sciences and Technology of Kashmir, Srinagar, Kashmir, J&K 190025 India; 3 Division of Plant Pathology, FoH, Sher-e-Kashmir University of Agricultural Sciences and Technology of Kashmir, Srinagar, Kashmir, J&K 190025 India; 4 Division of Fruit Science, FoH, Sher-e-Kashmir University of Agricultural Sciences and Technology of Kashmir, Srinagar, Kashmir, J&K 190025 India; 5 Petroleum and Chemical Engineering, Faculty of Engineering, Universiti Teknologi Brunei, Bandar Seri Begawan, BE1410 Brunei Darussalam

**Keywords:** Biological techniques, Biotechnology, Microbiology, Plant sciences

## Abstract

The less phytopathogen susceptibility in Himalayan *Brassica rapa* L. has made it an exceptional crop eluding synthetic pesticide inputs, thereby guarantying economically well-founded and ecologically sustainable agriculture. The relevance of niche microflora of this crop has not been deliberated in this context, as endosymbiosiome is more stable than their rhizosphere counterparts on account of their restricted acquaintance with altering environment; therefore, the present investigation was carried out to study the endophytic microfloral dynamics across the *B*. *rapa* germplasm in context to their ability to produce chitinase and to characterize the screened microflora for functional and biochemical comportments in relevance to plant growth stimulation. A total of 200 colonies of bacterial endophytes were isolated from the roots of *B*. *rapa* across the J&K UT, comprising 66 locations. After morphological, ARDRA, and sequence analysis, eighty-one isolates were selected for the study, among the isolated microflora *Pseudomonas* sp. *Bacillus* sp. dominated. Likewise, class γ-proteobacteria dominated, followed by Firmicutes. The diversity studies have exposed changing fallouts on all the critical diversity indices, and while screening the isolated microflora for chitinase production, twenty-two strains pertaining to different genera produced chitinase. After carbon source supplementation to the chitinase production media, the average chitinase activity was significantly highest in glycerol supplementation. These 22 strains were further studied, and upon screening them for their fungistatic behavior against six fungal species, wide diversity was observed in this context. The antibiotic sensitivity pattern of the isolated strains against chloramphenicol, rifampicin, amikacin, erythromycin, and polymyxin-B showed that the strains were primarily sensitive to chloramphenicol and erythromycin. Among all the strains, only eleven produced indole acetic acid, ten were able to solubilize tricalcium phosphate and eight produced siderophores. The hydrocyanic acid and ammonia production was observed in seven strains each. Thus, the present investigation revealed that these strains could be used as potential plant growth promoters in sustainable agriculture systems besides putative biocontrol agents.

## Introduction

Plants interact with an eclectic array of bacterial strains having a neutral, pathogenic, or beneficial influence on their hosts in both the usual and managed niches^1^. Most of the symbiotic microbial entities known as epiphytes take over the rhizosphere^2^. But a subclass of bacteria from the rhizosphere are proficient enough to enter and flourish inside the plant as endophytes and establish a mutualistic association^3^. These endosymbiotic assemblages are universal and well known to dwell inside the plant endosphere without instigating any detrimental upshot to the host^4^. In many investigations worldwide, endophytic bacterial strains have been isolated from virtually all the plants and plant parts such as roots, leaves, barks, stems, floral organs, and seeds^5–7^. To uphold a stable relationship with the host, these endophytic bacterial strains have ceaselessly evolved with the plants across the family lines to ameliorate it and endure biotic and abiotic stress through multiple direct and indirect mechanisms^6^. Most of them have a significant bearing on the growth and metabolism of the plants and^8^ are metabolically versatile, bearing the population load relatively short to their pathogenic counterparts^9^. There has been a close association of microbes with the plants for a variety of reasons, such as growth elevation, improved yield, and disease control; such associations continue to happen for centuries and are driven by the dependence of the microbes for their survival on the host plants^10^. These endophytes have defined niches in the plants and modulate the growth of the host by discharging nutrients, the defense system inductions, and the second synthesis of metabolites^11,12^. They communicate information to the plant host through interactions and biological reactions that result in phytohormone production, such as indole acetic acid (IAA), gibberellins or)^13^, organic compounds^14^ allelopathy, as well as systemic acquired resistance against the diseases^15^. Apart from that, they enhance the plant’s ability to survive the pathogen proliferation through iron immobilization by siderophore production, hydrolytic enzyme production^16^, enrich the soil through phosphate solubilization^17^ and nitrogen fixation^18^, and produce a gamut of natural products that could be used as potential drugs in human wellbeing^19^. Plant endosymbiosis-oriented enzymes have substantial practical relevance in waste management and agriculture. The plant family *Brassicaceae* in the investigated region is least susceptible to phytopathogenic fungi but still has not been considered a vital source of agriculturally significant bacterial isolates, especially for the production of extracellular enzymes like chitinases, with the potential use in industry or as fungicides to control the growth of phytopathogenic fungi. Chitinolytic bacteria are a diverse functional group of bacteria with the ability to secrete extracellular chitinase that decomposes chitin, a bulky hydrophobic homopolymer chain consisting of repeated β-(1–4) linked N-acetyl-D-glucosamine units spread across the biological spectrum from fungal cell walls viz*.,* chitridiomycetes, basidiomycetes, ascomycetes and Deuteromycetes to insect exoskeleton and crustacean shells^20^. Each year, a considerable portion of chitin waste material is discharged into the environment leading to grave environmental apprehensions^21^. Chitin based cell wall degrading nature of these enzymes has made them a unique tool in regulating the generation of fungal protoplasts needed in the various research programs of biological origin^22^.

*Brassica rapa* (brown Sarson) belongs to the family *Brassicaceae* favors robust endophyte diversity within roots because reasonably good sulfur requirements are well-founded^2^. This root-associated microflora could fetch superior growth elevation and phytopathogen containment by synthesizing bio-chemicals and active compounds. Because of the elevated temperatures and significant climate change in the North-Western Himalayan zone, the crops would shortly be subjected to biotic and abiotic stress in challenging pathogenic strains and meager nutrient availability, respectively^1^. Under these circumstances, bacterial assemblages, on account of their more comprehensive metabolic flexibility, could play a more significant role in shaping the adaptableness of the host. Still, endophytes are more competent than their rhizosphere counterparts among the bacterial microflora because their diverse and well-founded anabolic pathways^18^ were considered for this study.

Plant communities in the North Western Himalayan habitat are controlled by the interactions between biotic and physicochemical apparatuses of the extreme climate matrix. Interactions with microbes appear decisive in disease-free crop standing and obtaining inorganic nutrients or growth-influencing substances. Despite the significant role of bacterial diversity in such plant communities, little is known about the distribution and richness of endophytic bacteria in *B. rapa* plant growing in such habitat. *Brassica rapa* plant has been studied concerning its various genetic, agronomic and physiological characteristics. However, no study has reported the relationships between the *B. rapa* plant and associated endophytic bacterial microflora. This study aimed to isolate and characterize the bacterial endosymbiosiome of *B. rapa* germplasm spread across the Noth Western Himaliayan niches, its diversity and chitinase production potential to assess the isolated microflora for plant growth-promoting (PGP) traits like production of IAA and siderophore, and phosphate solubilization. This study is the first report on the diversity of culturable endophytic bacteria associated with the *B. rapa* plant growing in this temperate habitat of the Himalayas. The outcome of this investigation shall not only add knowledge to the diversity studies. Still, it would also form a larger plinth for selecting endophytic bacteria that can be utilized to facilitate plant growth in such habitats. The studies on stress tolerance would pave a way forward to mitigate climate change mediated drought and elevated temperature stress. This study was aimed to visualize the bacterial endosymbiosiome structure and dynamics of chitinase producing microflora in *B. rapa* and to test the hypothesis that these bacterial entities, besides having an industrial potential in the context of chitinase production, are metabolically and biochemically diverse concerning their potential for plant growth promotion and disease mitigation.

## Results

### Physio-biochemical characterization

**Figure 1:**
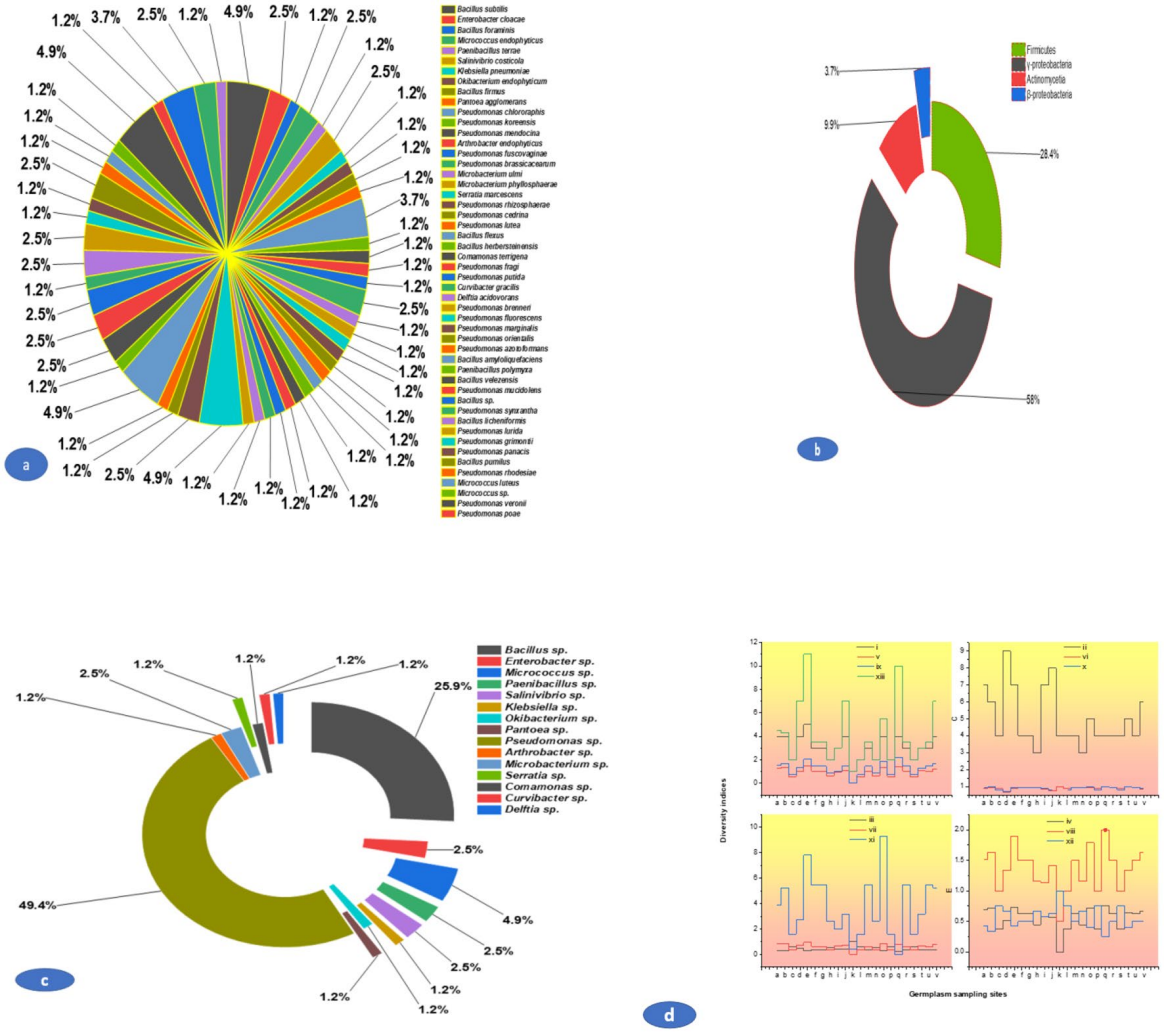
Structure of culturable bacterial endosymbiosiome associated with *B. rapa* L.; (**a**) Relative proportion of (species level); (**b**) Relative proportion of (genus level); (**c**) Relative proportion of the taxonomic classes; (**d**) Diversity indices of bacterial endosymbiosiome where symbols: (i) Taxa_S; (ii) Individuals; (iii) Dominance_D; (iv) Simpson_1-D; (v) Shannon_H; (v) Evenness_ e^H/S; (vii) Brillouin; (viii) Menhinick Margalef; (ix) Equitability_J; (x); (xi) Fisher_alpha; (xii) Berger-Parker and (xiii) Chao-1 and similarly the letters inside figures represent different sampling sites: (a)Akura; (b) Bona Nambal; (c) Hugam; (d) Hutmara; (e) Panzmulla; (f) Rakh Chandipora; (g) Tujar; (h) Bomai; (i) Brath; (j) Juhama; (k) Kanispora; (l) Fateh Pora; (m) Nowgam; (n) Rawalpora; (o) Rangreth; (p) Khunmoah; (r) Zakura; (s) Gulab Bagh; (t) Ahmad Nagar; (u) Dhara; (v) Tailbal and (w) Batapora.

A total of 200 colonies were isolated from the germplasm (roots) of *B. rapa* across the North-Western Himalayan zone of India (J&K) based on morphology (texture, color, elevation, margin, etc.). After ARDRA analysis, 81 strains were selected for this study. However, no colonies were on sterilized uncut roots close to the surface; the selected bacterial strains belonged to various sampling sites (Table S1). Out of the total of 81 isolated strains (Fig. 1a, Table S2) *Pseudomonas* sp. (n = 40, 49.38%) dominated followed by *Bacillus* sp. (n = 23, 28. 39%) (Fig. 1b), likewise, class γ-proteobacteria dominated (n = 47, 58.02%) followed by Firmicutes (n = 23, 28.39%) (Fig. 1c). The isolated bacterial strains showed a wide diversity across the germplasm collected from various sites. The Dominance diversity index was highest in Kansipora germplasm (1) and lowest in germplasm collected from Zakura (0.25), Simpson diversity index was 0 to 0.6939 in Kansipora and Akura, respectively. Similarly, the Shannon index was observed to be 0 and 1.475 in the sampled germplasm of Kansipora and Panzmulla, respectively. The Margalef index was 2.164 and 0 in the sampled germplasm from Zakura and Kansipora, respectively, similar to all other diversity indices [Brillouin, Menhinick, Fisher_alpha, etc.] varied widely among the isolated microflora (Fig. 1d).

**Figure 2:**
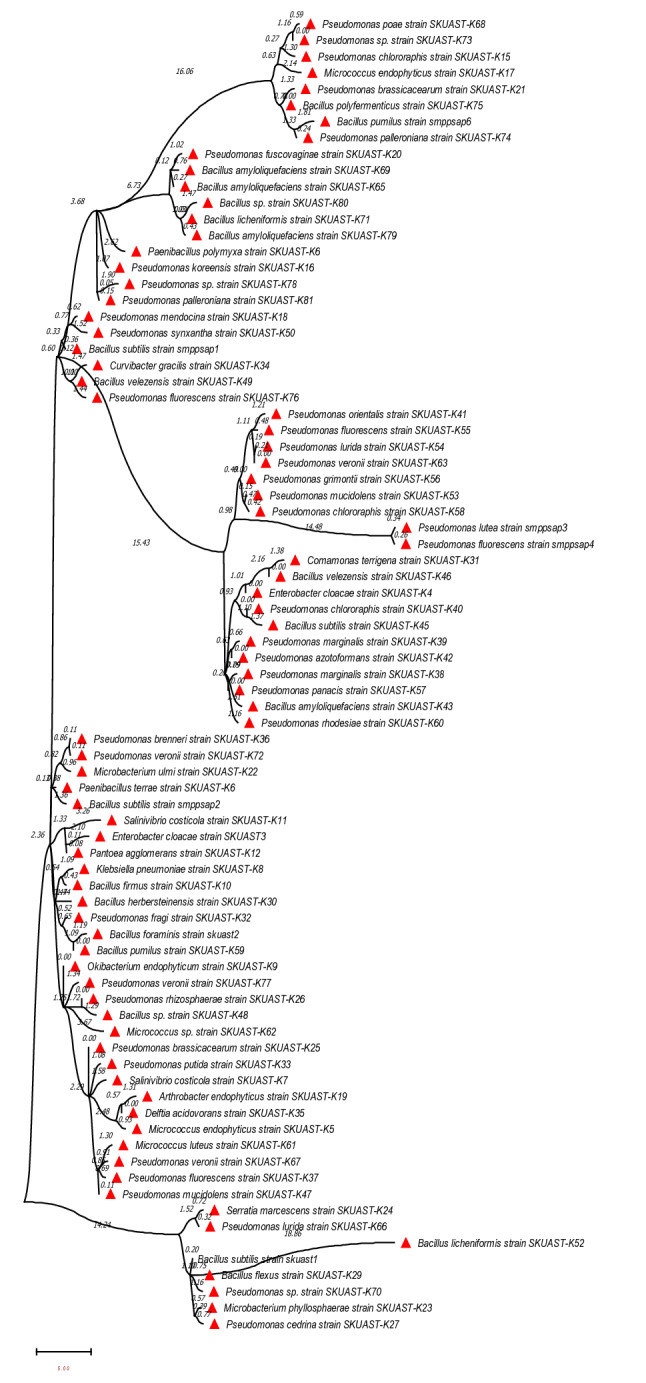
The evolutionary history was anecdotal via the Neighbor-Joining method. The ideal tree with the sum of branch length = 26.06144331 is shown. The bootstrap consensus tree anecdotal from 1000 replicates is kept to and divisions consistent to partitions replicated in less than 50 per cent bootstrap replicates are warped. The evolutionary detachments were computed by means of the p-distance method and are in the units of the number of base variances per site. The confidence likelihood that the interior branch length is larger than 0, as projected using the bootstrap test (revealed next to the kindling). This analysis involved 81 nucleotide sequences. Codon sites encompassed were 1st + 2nd + 3rd + Noncoding. All sites with fewer than 95% site coverage were rejected, i.e., ambiguous bases were allowed at any position (partial deletion option), missing data and fewer than 5% alignment gaps. There were a total of 1427 positions in the final dataset. Evolutionary studies were conducted in MEGA X (v 10.2.6).

**Figure 3:**
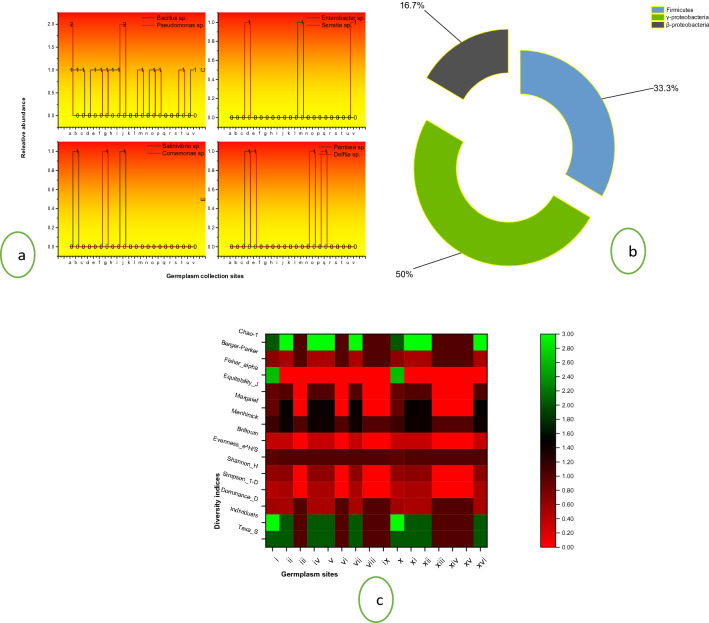
Relative proportion of chitinase producing bacterial endosymbiosiome associated with *B. rapa* L.; **(a)** genus level; (**b**) taxonomic classes and (**c**) Diversity indices of bacterial endosymbiosiome of *B. rapa* L. from different sampling sites, where symbols: (i) Akura; (ii) Bona Nambal; (iii) Hugam; (iv) Hutmara; (v) Panzmulla; (vi) Rakh Chandipora; (vii) Tujar; (viii) Bomai; (ix) Brath; (x) Juhama; (xi) Nowgam; (xii) Rangreth; (xiii) Khunmoah; (xiv) Zakura; (xv) Dhara; and (xvi) Batapora.

The phylogenetic relationship among the isolated strains is represented in Fig. 2. A total of 22 chitinase-producing strains belonging to eight genera were further studied in context to their diversity in the collected samples and plant growth-promoting potential; among these strains, *Bacillus* sp. dominated, followed by *Pseudomonas* sp. (Fig. 3a) likewise, class γ-proteobacteria dominated (Fig. 3b). The chitinase producing bacterial strains also showed a wide diversity; the Dominance diversity index was observed to vary from 0.5 to 1, the Simpson diversity index varied from 0 to 0.5, the Shannon index was observed to be varying from 0 to 0.693, Margalef index was as high as 1.443 and as low as 0 in the sampled germplasm, in the similar way all other diversity indices [Brillouin, Menhinick, Fisher_alpha, etc.] varied widely among the isolated microflora (Fig. 3c). The population of bacterial endophytes in the sampled tissues varied from Log_10_ 8.386 × 10^5^ to Log_10_ 4.227 × 10^5^ (CFU/g FW) at Hugam and Nowgam, respectively (Table S1).

**Figure 4:**
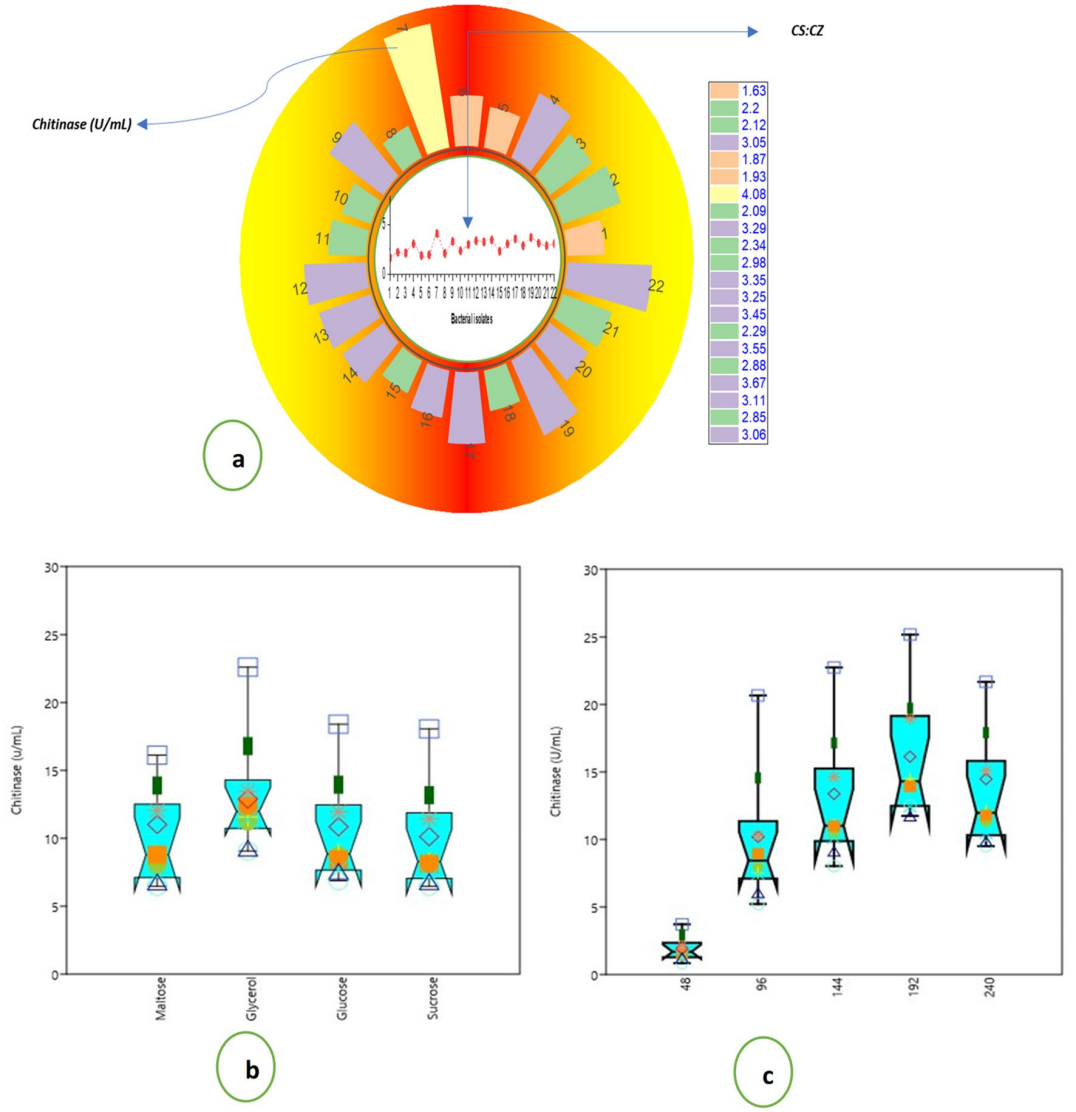
Magnitude of chitinase production among the isolated microflora; (**a**) chitinase activity (U/mL) of the isolated bacterial strains and their respective colony size to clearance zone ratio.**; (b)** Variation of chitinase production (U/mL) with sole carbon source viz., maltose, glycerol, glucose and sucrose; (**c**) Incubation period viz., 48, 96, 144, 192, and 240 h.

In the quantitative estimation of the chitinase production experiment, the highest chitinase production was observed in isolate smppsap2 (33.34 units/mL), while SKUAST-K31 was observed to produce the least (9.04 units/mL) chitinase. The CS: CZ ratio varied from 4.08 in strain smppsap2 to 1.63 in skuast2 (Fig. 4a). The species of *Bacillus (9), Pseudomonas (7), Salinivibrio (1), Pantoea (1), Comamonas (1), Serratia. (1), Delftia, (1), and Enterobacter*, based on their morphology and biochemical properties, have been allocated to respective lineages (Table 1). Based on the ability to produce chitinase, our investigation was restricted to these twenty-two bacterial strains, and their 16S rRNA gene sequence data are presented in (Table 2). Based on 16S rRNA gene sequences of the chitinase-producing microflora, the details of their closest neighbor are given in Table 3.

**Table 1:** Physio-biochemical attributes of the isolated microflora.

	*1*	2	*3*	*4*	*5*	6	7	8	*9*	10	*11*	12	13	14	15	*16*	*17*	*18*	*19*	*20*	*21*	*22*
I	−	−	−	−	−	−	−	−	−	−	−	−	−	−	−	−	−	−	−	−	−	−
II	+ve	−ve	+ve	−ve	−ve	+ve	+ve	−ve	−ve	−ve	−ve	−ve	+ve	+ve	+ve	+ve	−ve	−ve	−ve	−ve	+ve	−ve
III	R	R	R	R^$^	R	R	R	R	R	R	R	R	R	R	R	R	R	R	R	R	R	R
IV	C	−	C	−	−	C	C	−	−	−	−	−	−	T	C	C	−	−	−	−	−	−
V	−	+	−	−	−	−	−	+	−	−	+	+	−	−	−	−	+	+	+	+	−	+
VI	−	−	−	−	−	−	−	−	−	−	−	+	−	−	−	−	−	−	−	−	−	−
VII	−	−	−	−	−	+	−	−	+	−	−	−	−	−	−	−	−	−	−	−	−	−
VIII	+	−	+	−	−	+	+	−	−	−	−	−	+	−	+	+	−	−	−	−	+	−
IX	−	+	+	−	−	+	+	+	−	+	+	+	+	+	+	+	+	+	+	+	+	+
X	+	+	+	−	+	+	+	+	+	+	+	+	+	+	+	+	+	+	+	+	+	+
XI	−	−	−	−	−	−	−	−	−	−	−	−	−	+	−	−	−	−	−	−	−	−
XII	−	+	−	+	+	−	−	+	+	+	+	+	−	+	−	−	+	+	+	+	−	+
XIII	−	−	−	−	−	−	−	−	−	−	−	−	−	−	−	−	−	−	−	−	−	−
XIV	−	−	−	−	−	−	+	−	−	−	−	−	+	−	−	−	−	−	−	−	+	−
XV	+	+	+	+	+	+	+	+	+	+	+	+	+	+	+	+	+	+	+	+	+	+
XVI	+	+	+	+	+	+	+	+	+	+	+	−	+	+	+	+	+	+	+	+	+	+
XVII	−	+	−	+	−	−	−	−	−	+	+	−	+	+	+	+	+	+	+	+	+	+
XIX	+	+	+	+	+	+	+	+	+	+	+	−	+	+	+	+	+	+	+	+	+	+

1: *Bacillus foraminis* strain skuast2; 2: *Enterobacter cloacae* strain SKUAST-K4; 3: *Bacillus firmus* strain SKUAST-K10; 4: *Salinivibrio costicola* strain SKUAST-K11; 5: *Pantoea agglomerans* strain SKUAST-K12; 6: *Bacillus subtilis* strain smppsap1; 7: *Bacillus subtilis* strain smppsap2; 8: *Pseudomonas* koreensis strain SKUAST-K16; 9: *Serratia marcescens* strain SKUAST-K24; 10: *Comamonas terrigena* strain SKUAST-K31; 11: *Pseudomonas fragi* strain SKUAST-K32; 12: *Delftia acidovorans* strain SKUAST-K35; 13: *Bacillus amyloliquefaciens* strain SKUAST-K43; 14: *Bacillus subtilis* strain SKUAST-K45; 15: *Bacillus velezensis* strain SKUAST-K46; 16: *Bacillus velezensis* strain SKUAST-K49; 17: *Pseudomonas synxantha* strain SKUAST-K50*; 18: Pseudomonas fluorescens* strain smppsap4; 19*: Pseudomonas mucidolens* strain SKUAST-K53; 20: *Pseudomonas lurida* strain SKUAST-K54; 21: *Bacillus pumilus* strain smppsap6; 22: *Pseudomonas veronii* strain SKUAST-K67I : Pigment production;II: Gram reaction;III: Shape; IV: Endospore position; V: Indole production;V1: Methyl red test; VII: Voges-Proskauer reaction;V111: Citrate utilization; IX: Oxidase; X: Catalase; XI: H_2_S production; XII: Starch hydrolysis; XIII: Cellulose hydrolysis; XIV: Acid production; XV: Glucose; XVI: Sucrose; XVII: Lactose; XIX: Maltose; R: rods; $: Curved rods; C = Central; T = Terminal.

**Table 2:** Bacterial strains as identified on 16S rRNA gene sequencing.

Isolate	Accession number	Strain	Gene size amplified (bp)
SB2	KY548645	*Bacillus foraminis* strain skuast2	1484
SB4	KY612281	*Enterobacter cloacae* strain SKUAST-K4	1471
SB10	KY612277	*Bacillus firmus* strain SKUAST-K10	1502
SB11	KY612278	*Salinivibrio costicola* strain SKUAST-K11	1542
SB12	KY612268	*Pantoea agglomerans* strain SKUAST-K12	1427
SB13	KU674947	*Bacillus subtilis* strain smppsap1	1527
SB14	KU883268	*Bacillus subtilis* strain smppsap2	1523
SB16	KY612283	*Pseudomonas koreensis* strain SKUAST-K16	1461
SB24	KY612275	*Serratia marcescens* strain SKUAST-K24	1565
SB31	KY621796	*Comamonas terrigena* strain SKUAST-K31	1482
SB32	KY621553	*Pseudomonas fragi* strain SKUAST-K32	1504
SB35	KY621551	*Delftia acidovorans* strain SKUAST-K35	1533
SB43	KY646015	*Bacillus amyloliquefaciens* strain SKUAST-K43	1481
SB45	KY646007	*Bacillus subtilis* strain SKUAST-K45	1495
SB46	KY646014	*Bacillus velezensis* strain SKUAST-K46	1483
SB49	KY646001	*Bacillus velezensis* strain SKUAST-K49	1573
SB50	KY646008	*Pseudomonas synxantha* strain SKUAST-K50	1589
SB51	KU883270	*Pseudomonas fluorescens* strain smppsap4	1502
SB53	KY646003	*Pseudomonas mucidolens* strain SKUAST-K53	1475
SB54	KY646013	*Pseudomonas lurida* strain SKUAST-K54	1480
SB64	KU883601	*Bacillus pumilus* strain smppsap6	1526
SB67	KY646028	*Pseudomonas veronii* strain SKUAST-K67	1520

**Table 3:** Molecular facets of isolated microflora based on data retrieved from National Center for Biotechnology Information, USA.

Source	Strain	Neared phylogenetic neighbor	Similarity (%)	Gene size amplified (bp)	Accession number
*Bacillus foraminis*	skuast2	*Bacillus foraminis*	99.93	1484	KY548645
*Enterobacter cloacae*	SKUAST-K4	*Enterobacter cloacae*	99.80	1471	KY612281
*Bacillus firmus*	SKUAST-K10	*Bacillus firmus*	97.01	1502	KY612277
*Salinivibrio costicola*	SKUAST-K11	*Salinivibrio costicola*	99.73	1542	KY612278
*Pantoea agglomerans*	SKUAST-K12	*Pantoea agglomerans*	98.30	1427	KY612268
*Bacillus subtilis*	smppsap1	*Bacillus subtilis*	99.18	1527	KU674947
*Bacillus subtilis*	smppsap2	*Bacillus subtilis*	98.03	1523	KU883268
*Pseudomonas koreensis*	SKUAST-K16	*Pseudomonas koreensis*	100.0	1461	KY612283
*Serratia marcescens*	SKUAST-K24	*Serratia marcescens*	98.88	1565	KY612275
*Comamonas terrigena*	SKUAST-K31	*Comamonas terrigena*	98.90	1482	KY621796
*Pseudomonas fragi*	SKUAST-K32	*Pseudomonas fragi*	98.40	1504	KY621553
*Delftia acidovorans*	SKUAST-K35	*Delftia acidovorans*	97.78	1533	KY621551
*Bacillus amyloliquefaciens*	SKUAST-K43	*Bacillus amyloliquefaciens*	98.77	1481	KY646015
*Bacillus subtilis*	SKUAST-K45	*Bacillus subtilis*	99.02	1495	KY646007
*Bacillus velezensis*	SKUAST-K46	*Bacillus velezensis*	96.70	1483	KY646014
*Bacillus velezensis*	SKUAST-K49	*Bacillus velezensis*	96.63	1573	KY646001
*Pseudomonas synxantha*	SKUAST-K50	*Pseudomonas synxantha*	98.42	1589	KY646008
*Pseudomonas fluorescens*	smppsap4	*Pseudomonas fluorescens*	96.72	1502	KU883270
*Pseudomonas mucidolens*	SKUAST-K53	*Pseudomonas mucidolens*	96.95	1475	KY646003
*Pseudomonas lurida*	SKUAST-K54	*Pseudomonas lurida*	96.69	1480	KY646013
*Bacillus pumilus*	smppsap6	*Bacillus pumilus*	99.25	1526	KU883601
*Pseudomonas veronii*	SKUAST-K67	*Pseudomonas veronii*	98.00	1520	KY646028

### Chitinase production

Based on their ability to produce chitinase, the top ten isolates were further studied to make chitinase when augmented with various carbon sources (maltose, glycerol, glucose, and sucrose) supplementations under different incubation periods (Fig. 4b). The average chitinase activity was significantly highest in glycerol supplementation, followed by glucose. The minor chitinase activity was observed in maltose. The incubation period greatly influenced chitinase activity, with the average highest chitinase activity at 192 h followed by 240-h incubation (Fig. 4c). With the increase in the incubation period, there was a corresponding increase in chitinase activity, and maximum activity was observed at the 192-h incubation period, beyond which a decline was noticed. The significantly most minor average chitinase activity among all the incubations was honored at the incubation period of 48 h, and all the isolates followed this trend. The chitinase production by all the isolates increased significantly with the incubation period from 48 to 192 h; there was a decrease beyond 192 h incubation.

### Indole-3-acetic acid and other plant growth associated metabolite production

Among all the strains, only eleven produced IAA, and the highest production was observed in smppsap6 (16.55 µg/mL) and the lowest (3.13 mg/mL) in skuast2 strain (Table 4). A total of ten strains were able to solubilize tricalcium phosphate varying from 3.07 to 9.04 mm; the solubilization index ranged from 1.24 in strain SKUAST-K11 to 2.53 in strain SKUAST-K43. The phosphorus released expressed as mg/L varied from 38.23 in strain SKUAST-K67 to 264.80 mg/L in strain SKUAST-K46. The siderophore production expressed as percent unit was produced by eight strains and varied from 9.93 in strain SKUAST-K35 to 18.83 in smppsap4 strain. The HCN production described as absorbance (λ_625_ nm) was created by seven strains and ranged from 0.012 in strain SKUAST-K46 to 0.217 in strain smppsap4. Similarly, ammonia production was observed in seven isolates and ranged from 9.14 in strain SKUAST-K54 to 52.17 in strain SKUAST-K50 (Table 4).

**Table 4:** Plant growth-promoting attributes of the isolated microflora.

Strains	IAA	Solubilization zone (mm)	Solubilization index	P-released (mg/L)	(% Siderophore unit)	HCN (λ_625_ nm)	Ammonia (µg/mL)
skuast2	3.13^j^	–	–	–	–	–	–
SKUAST-K4	–	7.05^b^	2.17^c^	105.75^b^	–	–	–
SKUAST-K10	–	5.06^g^	2.04^d^	93.69^d^	–	–	–
SKUAST-K11	–	3.04^j^	1.24^h^	63.75^h^	–	–	–
SKUAST-K12	9.72^e^	–	–	–	–	–	37.07^b^
smppsap1	9.55f.	–	–	–	17.26^d^	0.101^b^	–
smppsap2	11.84^b^	–	–	–	14.18f.	0.087^c^	–
SKUAST-K16	–	–	–	–	12.22^g^	–	–
SKUAST-K24	–	–	–	–	–	–	–
SKUAST-K31	7.65^i^	5.53^d^	2.52^b^	91.25^b^	–	–	–
SKUAST-K32	8.17^g^	7.04^c^	1.90f.	101.5^**c**^	–	–	–
SKUAST-K35	–	–	–	–	9.93^h^	–	–
SKUAST-K43	9.36^g^	5.51^e^	2.53^a^	92.50^c^	–	0.019f.	19.18f.
SKUAST-K45	–	–	–	–	–	–	24.07^c^
SKUAST-K46	5.92^i^	9.04^a^	2.52^b^	264.80^a^	14.89^e^	0.012^g^	–
SKUAST-K49	–	–	–	–	17.86^b^	–	–
SKUAST-K50	–	5.33^b^	1.85^**e**^	91.12^g^	–	–	52.17^a^
smppsap4	11.05^c^	–	–	–	18.83^a^	0.217^a^	22.07^d^
SKUAST-K53	–	–	–	–	–	–	–
SKUAST-K54	–	–	–	–	–	–	9.14^g^
smppsap6	16.55^a^	4.23^h^	1.85^e^	42.50^i^	–	0.065^c^	21.67^e^
SKUAST-K67	10.11^d^	3.07^i^	1.37^g^	38.23^j^	17.51^c^	0.069^d^	–

Means in the column with same letter didn’t differ by Tukey’s t-test.

### Antifungal activity

**Figure 5:**
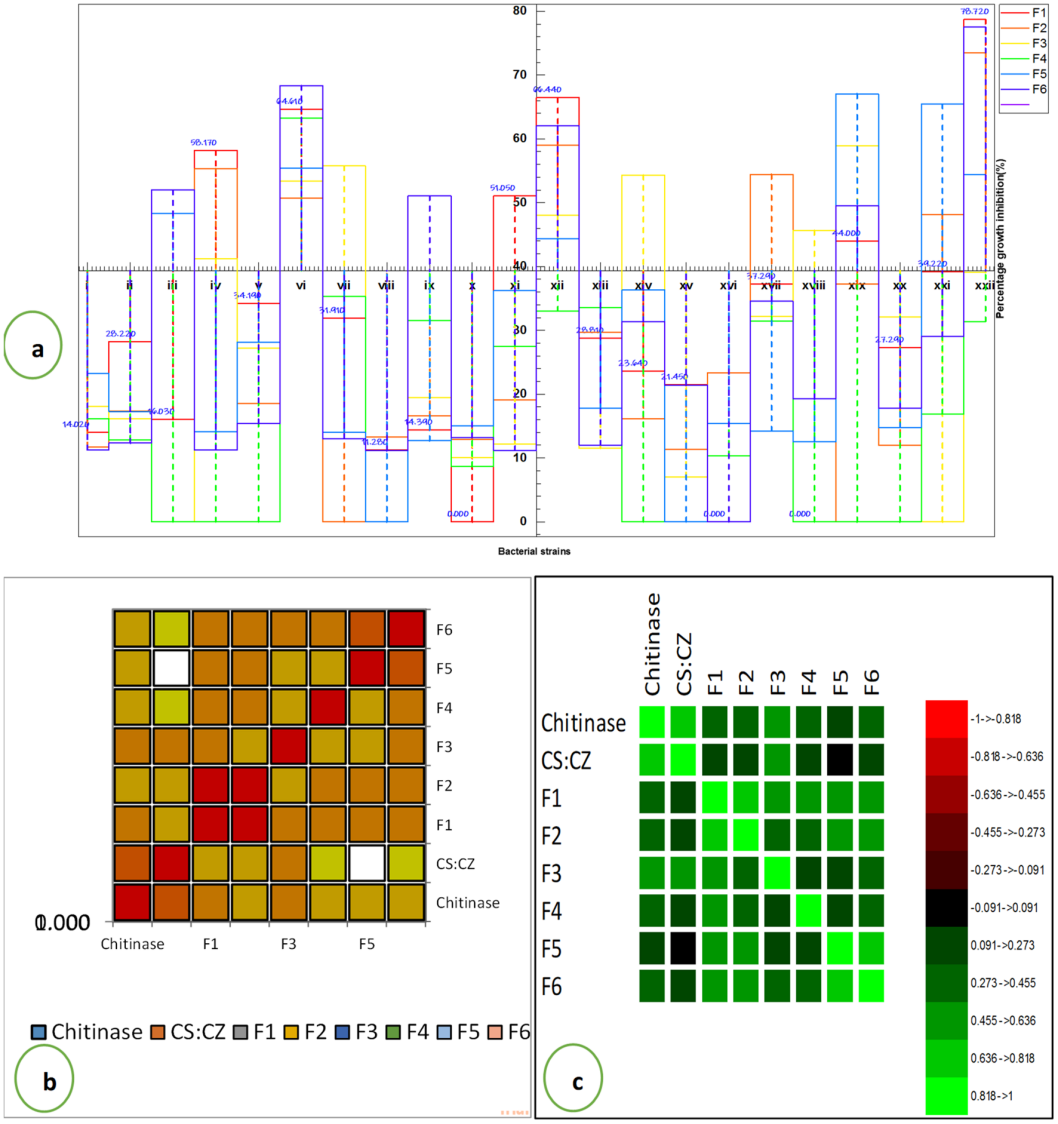
**(a)** Isolated microflora representing fungicidal activity expressed as percentage inhibition of; F1: *Fusarium oxysporum*; F2; *F. solani*; F3: *Dematophora. necatrix*^1^; F4: *Pythium amphanidermatum*; F5: *D. necatrix*^2^ and F6: *Rhizoctonia solani*. where symbols: (i) Akura; (ii) Bona Nambal; (iii) Hugam; (iv) Hutmara; (v) Panzmulla; (vi) Rakh Chandipora; (vii) Tujar; (viii) Bomai; (ix) Brath; (x) Juhama; (xi) Kanispora; (xii) Fateh Pora; (xiii) Nowgam; (xiv) Rawalpora (xv) Rangreth; (xvii) Khunmoah; (xvii) Zakura; (xviii) Gulab Bagh; (xix) Ahmad Nagar (xx) Dhara; (xxi) Tailbal and (xxii) Batapora.; (**b**) Correlation map and (c) Correlation matrix pertaining to chitinase; CS: CZ and antifungal behavior against F1 (*F. oxysporum*): F2 (*F. solani*); F3 (*D. necatrix*^1^; F4 (*P. amphanidermatum*); F5 (*D. necatrix*^2^) and F6 (*Rhizoctonia solani*).

To assess the fungistatic property of the bacterial strains, six fungal species, namely *Fusarium oxysporum*, *Fusarium solani*, *Dematophora necatrix*^*1*^, *Dematophora necatrix*^*2*^ *Rhizoctonia solani,* and *Pythium amphanidermatum,* were used, and fungistatic activity was expressed by calculating percent growth inhibition. All the strains exhibited fungistatic activity (Fig. 5a). A total of 19 isolates were found to be fungistatic against *F. oxysporium,* and the percentage growth inhibition ranged from 11.28 to 78.72 percent. Similarly, 19 isolates were observed to be fungistatic against *F. solani.* The percentage growth inhibition ranged from 11.39 to 59.05 percent; 21 isolates were honored to be fungistatic against *R. solani.* The percentage growth inhibition ranged from 11.15 to 77.54 percent; 13 isolates were observed to be fungistatic against *P. amphanidermatum.* The percentage growth inhibition ranged from 8.66 to 63.23 percent. 19 isolates were honored to be fungistatic against *D. nectrix* strain 1, and the percentage growth inhibition ranged from 7.04 to 58.90 percent. A total of 20 isolates were found to be fungistatic against *D. nectrix* strain 2, and the percentage growth inhibition ranged from 12.55 to 67.06 percent (Fig. 5a). The correlation studies represented by both matrix and map in Fig. 5b,c depict that chitinase activity and antifungal behavior in most isolated strains seem to be positively correlated.

### Antibiotic sensitivity

**Figure 6:**
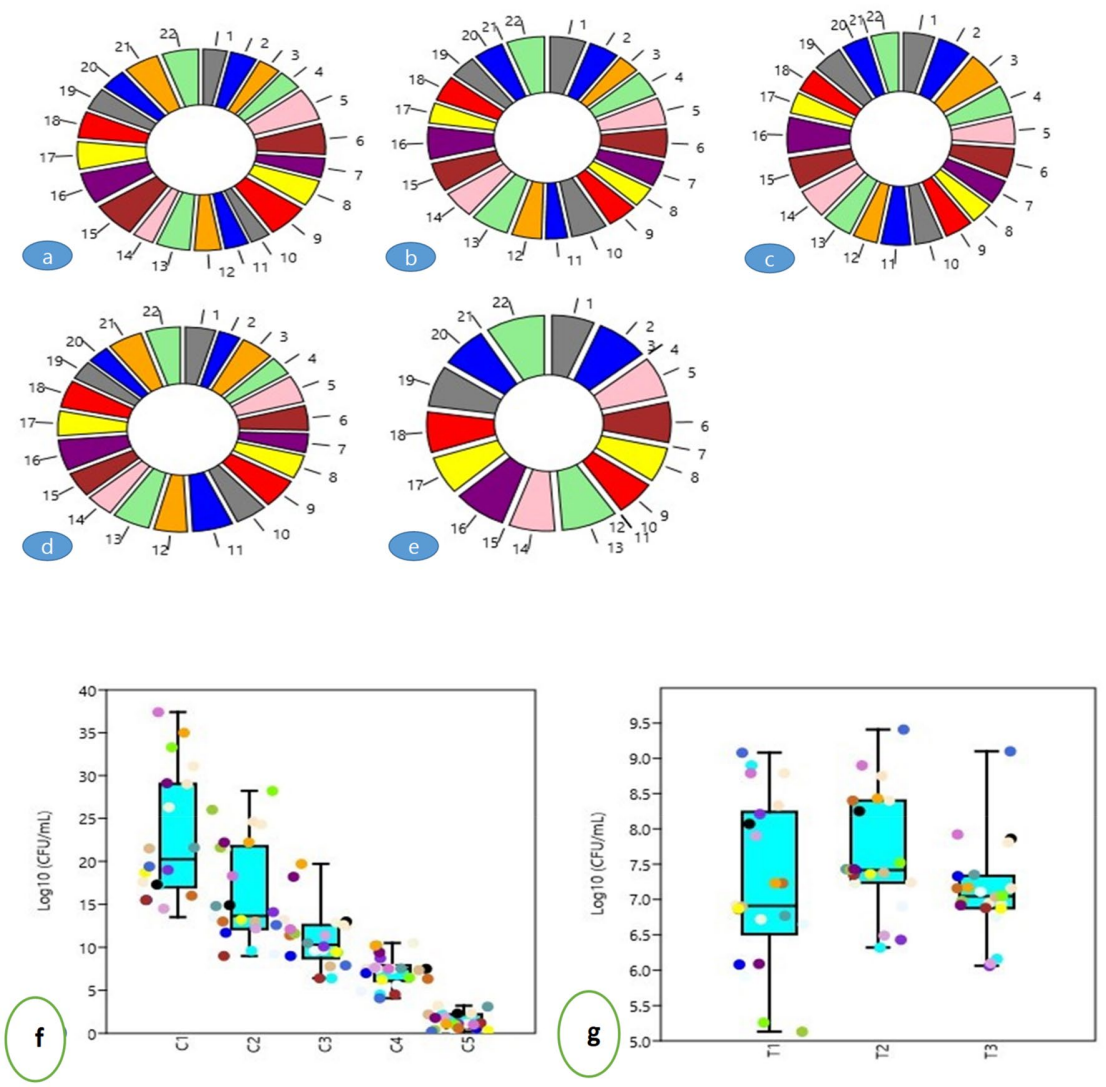
Representation of antibiotic sensitivity of the isolated microflora against [inhibition zone (mm)] (**a**) Chloramphenicol; (**b**) Rifampicin; (**c**) Amikacin; (**d**) Erythromycin and (**e**) Polymyxin-B, the numbers represent: 1: *Bacillus foraminis* strain skuast2; 2: *Enterobacter cloacae* strain SKUAST-K4; 3: *Bacillus firmus* strain SKUAST-K10; 4: *Salinivibrio costicola* strain SKUAST-K11; 5: *Pantoea agglomerans* strain SKUAST-K12; 6: *Bacillus subtilis* smppsap1; 7: *Bacillus subtilis* smppsap2; 8: *Pseudomonas koreensis strain SKUAST-K16; 9: Serratia marcescens* strain SKUAST-K24; 10: *Comamonas terrigena* strain SKUAST-K31; 11: *Pseudomonas fragi* strain SKUAST-K32; 12: *Delftia acidovorans* strain SKUAST-K35; 13: *Bacillus amyloliquefaciens* strain SKUAST-K43; 14: *Bacillus subtilis* strain SKUAST-K45; 15: *Bacillus velezensis* strain SKUAST-K46; 16: *Bacillus velezensis* strain SKUAST-K49; 17: *Pseudomonas synxantha* strain SKUAST-K50; 18: *Pseudomonas fluorescens* strain smppsap4; 19: *Pseudomonas mucidolens* strain SKUAST-K53; 20*: Pseudomonas lurida* strain SKUAST-K54; 21: *Bacillus pumilus* strain smppsap6; 22: *Pseudomonas veronii* strain SKUAST-K67I; while as Box and Jitter representing (**g**) Variation of bacterial population (log_10_ CFU/mL) of the isolated microflora with different concentrations of PEG (C1: 0; C2 :10; C3: 15; C4 :20 and C5 :25% respectively). (**h**) Variation of bacterial population (log10 CFU/mL) of the isolated microflora with different temperature treatments (T1: 15; T2 : 25 and T3 : 35 °C), the symbols represent: Aliceblue: skuast2; Antiquewhite SKUAST-K4; Aqua: SKUAST-K10; Azure SKUAST-K11; Beige : SKUAST-K12; Bisque: smppsap1; Black : smppsap2; Blanchedalmond: SKUAST-K16; Blue : SKUAST-K24; Blueviolet: SKUAST-K31; Brown : SKUAST-K32; Burlywood: SKUAST-K35; Cadetblue : SKUAST-K43; Chartreuse :SKUAST-K45; Chocolate : SKUAST-K46; Yellowgreen : SKUAST-K49; Yellow : SKUAST-K50; Royalblue : smppsap4; Plum : SKUAST-K53; Purple : SKUAST-K54 : Orchid; smppsap6; Orange : SKUAST-K67.

Five antibiotics, such as chloramphenicol, rifampicin, amikacin, erythromycin, and polymyxin-B by Disc-Diffusion methods, have been accessed to investigate antibiotic susceptibility patterns in endophytic bacterial strains. Results shown in Fig. 6a–e indicate that *B. rapa* endophytic chitinase producing microflora was primarily vulnerable to rifampicin, erythromycin, polymyxin-B, and amikacin, followed by chloramphenicol and erythromycin. While as the resistance in the isolated strains was highest towards amikacin, followed by polymyxin-B and rifampicin, respectively. Among all the strains, none was found resistant to chloramphenicol and erythromycin.

### Stress tolerance

All the bacterial strains grew at all temperatures. At the temperature of 25 °C, the lowest growth was found in (Log_10_ 6.32 × 10^6^) in the SKUAST-K10 strain, whereas the highest growth of (Log_10_ 9.41 × 10^6^) was recorded in the smppsap4 strain against the control. At 35 °C temperature, the lowest growth was found (Log_10_ 6.10 × 10^6^) in SKUAST-K53 strain, and the highest increase was recorded (Log_10_ 9.10 × 10^6^) in smppsap4 strain, in contrast, to control (Fig. 6f). All the bacterial strains grew at all concentrations of PEG6000. At 25% PEG concentration, the smppsap4 showed the lowest (Log_10_ 0.2 × 10^6^), and SKUAST-K43 (Log_10_ 10.7 × 10^6^) had the highest population respectively, against the control (Fig. 6g).

### In vivo* studies*

**Figure 7:**
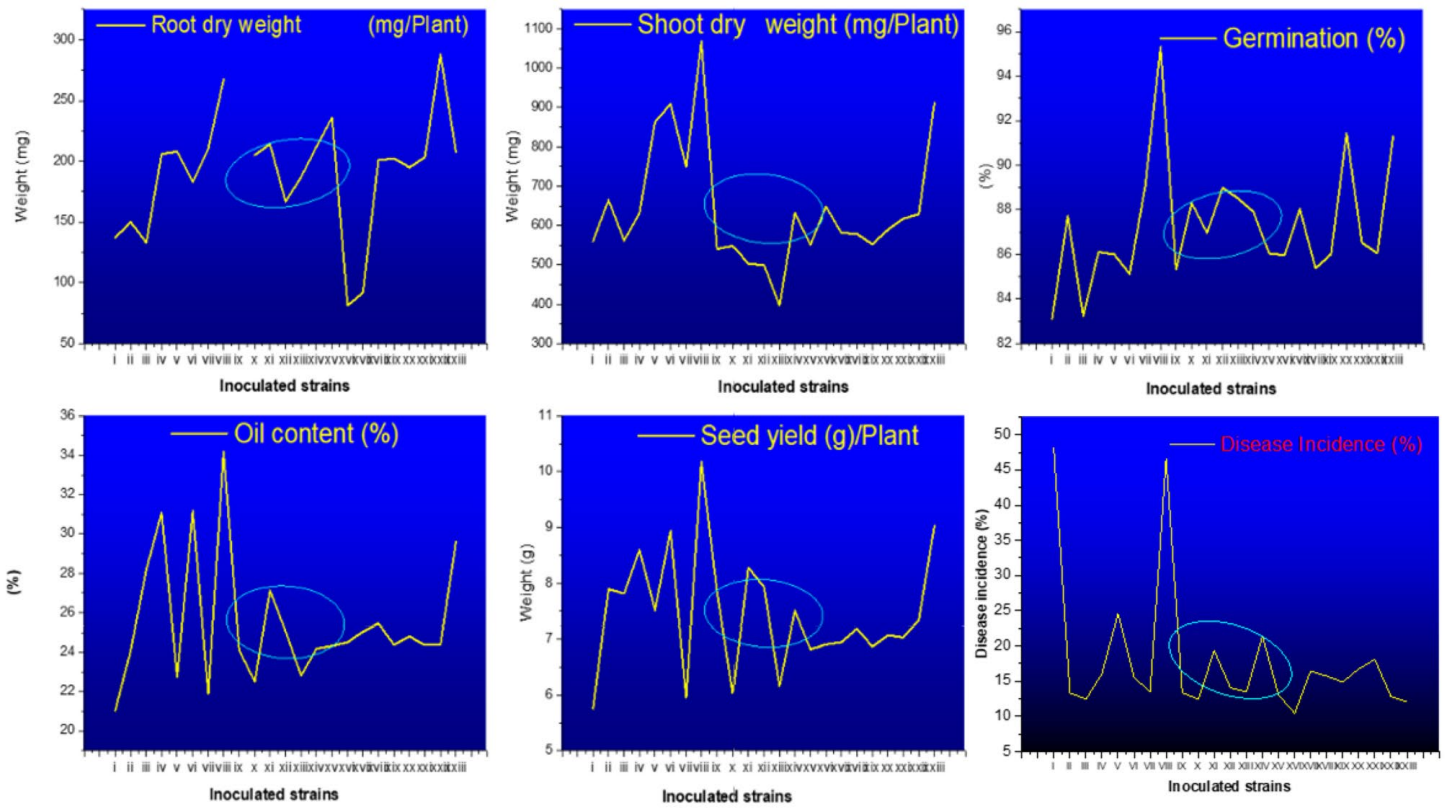
Effect of isolated microflora on growth and yield of *B. rapa* under pot house conditions. Symbols: *I:* Control; *II:* skuast2; *III:* SKUAST-K4; *IV:* SKUAST-K10; *V:* SKUAST-K11; *VI:* SKUAST-K12*; VII:* smppsap1; *VIII:* smppsap2; *IX:* SKUAST-K*16;* X: SKUAST-K24; XI: SKUAST-K31; XII: SKUAST-K32; XIII: SKUAST-K35; XIV: SKUAST-K43; XV: SKUAST-K45; *XVI:* SKUAST-K46; *XVII:* SKUAST-K49*; XVIII:* SKUAST-K50; *XIX:* smppsap4*; XX:* SKUAST-K53*; XXI:* SKUAST-K54*; XXII:* smppsap6*; XXIII:* SKUAST-K67I.

The inoculation experiments under pot house conditions using *B. rapa* as a host revealed that all the bacterial strains caused a considerable increase in both growths, yield and disease containment of the host plant (Fig. 7). The germination percentage was highest observed in the smppsap2 inoculated host (95.31 ± 0.21) against the control (83.12 ± 0.05); all the isolates caused a significant increase in root and shoot dry weights with the highest source and shoot dry weight (mg/plant) in 267.18 ± 0.03 and 1068.01 ± 0.11 against the control with 137.21 ± 0.41 and 561.02 ± 0.27 respectively. Similarly, the yield per plant and oil content (%) were significantly elevated in all the inoculated plants. The oil content (%) and seed yield (g/plant) was highest in smmppsap2 inoculated plants (34.19 ± 0.18 and 10.18 respectively) against the control with an oil content of 21.04 ± 0.14 (%) and 5.76 ± 0.09 (g/plant) respectively. All the strains prevalently reduced the disease intensity in all the inoculated plants against the control. The disease intensity was as low as 10.44% in the SKUAST-K46 inoculated strain against the control (48.09%).

## Discussion

Over the past few decades, extensive use of chemical fertilizers, herbicides, and pesticides has raised environmental concerns to prevent food scarcity globally and escalate crop yield. It is one of the burning issues in agriculture and oilseed production^23^. It has become imperative to seek alternative means to contain these ecologically flawed stratagems. The beneficial bacteria that inhabit the plants can be instrumental in improving the growth of plants under both usual and challenging circumstances^24^. They can improve the plant nutrient uptake, amend the soil health, and prime plant defense to develop quality bio inoculants for a stable yield and sustainability in agriculture^1^. Hence, understanding the endophytic microfloral community associated with *B. rapa* has become significant, besides their role in facilitating plant growth. Plant growth-promoting bacteria proliferate the endosphere after rhizosphere colonization to endeavor beneficial effects on plant growth. Many hypotheses, including motility, plant-polymer degradation, production of phytohormones, solubilization of phosphates, enzyme production, siderophore production, and evasion of plant defenses, have been described as involved in growth promotion by these biological entities^25^. *B*. *rapa,* together with other family members, forms the world's second most important oilseed crop nutritionally; its protein profile is comparable with the soya bean and contains more S-amino acids than any other oilseed meal^26^. The huge diversity of the bacterial microflora in the Himalayan germplasm of the plants is attributed to Spatio-temporal scales besides the wide climate variation gradient stretched across the Himaliyas^1^. Because of this huge diversity on account of the above predisposing factors in the area, we investigated the endophytic bacterial microflora of the crop (*Brassica rapa*), and the dynamics of this microflora in chitinase producing ability as no literature about the crop is available in this context. The present investigation was undertaken to document the diversity of bacterial endosymbiosiome associated with this crop to fill this knowledge gap. The screening for chitinase production and the chitinase-producing microflora was further investigated for its potential to increase plant growth, disease containment, and stress tolerance. The sampling area was divided into three parts (Baramulla in the North, Srinagar in the center, and Anantnag in the south) depending on the presence of maximum germplasm footprints of *B. rapa* L. These three different sites were explored for collecting the samples to study the bacterial endosymbiosiome followed by its chitinase producing ability. The PCR amplification followed by 16S rRNA gene sequencing and phylogenetic analysis determined these isolates' molecular identity and genetic diversity. A similarity search using NCBI GenBank identified all the isolates showing 91–100% identity with reference sequences. The 16S rRNA gene sequencing is a robust technique for identifying bacteria at the genus level, differentiating between closely related bacterial species^27^. Out of the total of 81 isolated strains *Pseudomonas* sp. (n = 40, 49.38%) dominated followed by *Bacillus* sp. (n = 21, 25.92%), similarly class γ-proteobacteria (n = 47, 58.02%) dominated followed by Firmicutes (n = 23, 28.39%). The strains of *Bacillus* sp. and *Pseudomonas* sp. have long been described as endophytes in tomatoes and ginger rhizomes of many plant species, including the family Brassicaceae (Jiménez-Gómez et al., 2020)^28–30^. Many investigators have reported similar findings concerning the dominance of genera among isolated microflora (Jiménez-Gómez et al., 2020)^31–33^. All the collected microflora was visualized for diversity, and, in this context, the diversity indices*,* namely the Simpson index, Shannon index, Margalef index, and other indices, varied greatly. Investigations on the putative hyper-diversity indices concerning endophytes in all key linages of terrestrial plants have exposed changing fallouts on all the key diversity indices^34^. The niche proliferating communities may largely be inclined by the type of habitat of the plantations^35^, thereby confirming our findings on the examined facade of diversity. The population of bacterial endophytes in the sampled tissues varied from Log_10_ 8.386 × 10^5^ to Log_10_ 4.227 × 10^5^ (CFU/g FW), which agrees with the population arrays in preceding investigations of endophytic bacteria in many crops like tomato^36,37^. The variation in the bacterial endophytic populations experienced robust fluctuations that prevalently depended upon factors including the type of germplasm screened, the physiological transitions, the dynamics among the host plants, and the soil environment attributes such as nutrient dynamics, organic matter fluctuations, plant genetic constitution, tissue type, and plant ecology^38^.

While screening the microflora for chitinase production, the different strains comprised: *Bacillus* (9), *Pseudomonas* (7), *Salinivibrio* (1), *Pantoea* (1), *Comamonas* (1), *Serratia*. (1), *Delftia,* (1) and *Enterobacter* (1). The large biochemical diversity concerning indole production, H_2_S, catalase, cellulase, oxidase production, etc., observed in the isolated chitinase producing endosymbiotic microflora is in tone with the previous investigations^11^, Which reported the existence of thirty bacterial endophytes in pearl millet with varying degree of biochemical and morphological attributes. It could be attributed to studying bacteria's endosymbiotic niche colonization lifestyle^1^. Chitinases with similar activity ranges have been characterized in many endophytic microfloral strains of bacteria^39,40^; these strains produced chitinase as low as 0.5 U/mL up to as high as 30 U/mL, thereby supporting our findings strongly. In the present investigation, 22 endophytic bacterial root endophytes associated with *B. rapa* produced chitinase enzyme. The CS: CZ ratio varied from 1.63 to 4.08. The significance of bacterial endophytes delivering chitinase in a colloidal chitin-containing environment with similar CS: CZ has been previously investigated^41,42^, wherein the isolated microflora from multiple agronomic crop studies had the CS: CZ ratio ranging from 1–6. After optimizing various carbon source supplementations under 5 incubation periods, the average chitinase activity was significantly highest in glycerol supplementation and least in maltose. With the increase in the incubation period, there was a corresponding increase in chitinase activity, and maximum activity was observed during the 192-h incubation period. A decline was noticed at least at 48 h. Our findings indicate that bacterial endophyte chitinase is inducible and repressed by additional sugar catabolites. The increased chitinase production with an incubation period up to 192 h and then declines could be attributed to autolysis of the enzyme or catabolite repression when the threshold limit is reached; these findings are in line with various investigations^43,44^, although contrary to our findings there was huge variation among the bacterial strains, some showing peak production with maltose as well, which could be attributed to the diversity in the metabolism of the endophytic bacterial microflora. The chitinase producing bacterial strains also showed a wide variety; the Dominance diversity index was observed to vary from 0.5 to 1, the Simpson diversity index changed from 0 to 0.5, the Shannon index was honored to be ranging from 0 to 0.693, Margalef index was as high as 1.443 and as low as 0 in the sampled germplasm, in the similar way all other diversity indices [Brillouin, Menhinick, Fisher_alpha, etc.] varied widely among the isolated microflora. The variation in the diversity indices of chitinase-producing bacterial endophytic populations has been observed in a similar range. A similar magnitude of diversity indices viz., Dominance, Simpson diversity index, Brillouin, Menhinick, Fisher_alpha, and other indices were also reported in the endophytic strains of Himalayan origin Padder et al.^1^. It is attributed to selection pressure in disease escape^4^.

Of all the isolated strains, only eleven produced IAA, HCN, and ammonia production in seven isolates. Dey and Raghuwanshi^45^, while investigating the comprehensive assessment of growth parameters for screening endophytic bacterial strains in Solanum Lycopersicum, observed that IAA production varied from 8.818 to 67.636 μg/ml in the isolated strains. Similarly, HCN and ammonia production was detected among the strains. A total of ten endophytic strains were able to solubilize tricalcium phosphate; the solubilization index and phosphorous released in liquid assay varied considerably among the isolates. Dey and Raghuwanshi^46^ also observed the solubilization index as high as 3.714, and solubilization of tricalcium phosphate was caused by all the isolated endophytic bacterial strains except the one strain RR11. Phosphate solubilization has been observed in various endophytic bacteria strains in a similar range^47,48^.

Furthermore, it was observed that eight strains produced siderophores, Lipková et al.^49^ while visualizing the growth promotion and disease suppressing ability of endophytic strains of *Brassica napus* L. observed that the majority (37 out of 38) strains produced siderophores, thus validating our supporting our findings that siderophore producing endophytic bacterial strains exist in the brassicacae family. Iron-chelating microbes in complicated environments have made plants eligible for growth^46^. These plants have been found to provide appropriate niches to support the development of microflora^50^. The present investigation revealed that the isolated bacterial microflora behaved differently to the various antibiotics; it was observed that the isolated strains were primarily vulnerable to rifampicin, erythromycin, polymyxin-B, and amikacin, followed by chloramphenicol and erythromycin. The endophytic microflora has a massive diversity in context to antibiotic sensitivity. Even the candidates from the sole genus behave erratically to a specific antibiotic^51,52^. ^T^heir behavior towards antibiotics like rifampicin, erythromycin, polymyxin-B, and amikacin has been observed similarly earlier^53^. All isolated bacterial endophytes grew in induced drought and elevated temperature mediated stress. The endophytes have been reported to withstand elevated temperatures even as high as 55 °C as low as subzero by Padder et al.^1^. The stress tolerance in the isolated microflora has revealed the more significant role of these endosymbiosiome entities shortly in changing global climate scenarios^54–59^. The stress tolerance in the bacteria can be attributed to many factors, like temperature, water, and other indigenous factors of the bacterial niche from which they are isolated^60^. Therefore, the possible reason to stress tolerance in the isolated microflora of the present investigation could be that the isolation in the present study was done where the temperature goes as high as 35 °C in the active growing season of *B. rapa* and during winters, the frozen environment causes the water deficit conditions and as such the associated microflora has evolved to challenge this stress.

To assess the fungistatic property of the bacterial strains, six fungal species, namely *F. oxysporum*, *F. solani*, *D. necatrix*^*1*^, *D. necatrix*^*2*^, *R. solani,* and *P. amphanidermatum,* were used. All the strains exhibited fungistatic activity except strain smppsap3. 19, 19, 21, 13, 19, and 20 isolates were found fungistatic against *F. oxysporium*, *F. solani*, *R. solani, P. amphanidermatum, D. nectrix*^*1*^*,* and *D. nectrix*^*2*^*,* respectively. Endophytic microflora, predominantly the species of *Bacillus* and *Pseudomonas,* have been found to cause the percentage growth inhibition to the extent of 90% and more in dual culture experiments with many pathogens like *Fusarium* sp., *Pythium* sp. etc. and thus protect plants against various pathogens and increase seedling emergence^61^. ‘Antibiotic associations among fungi and bacteria are significant for soil and ecological functions^62^. The antifungal activity of the isolated strains can be attributed to lytic enzymes, chitinases, antibiotic synthesis, ammonia, and siderophores, among other secretory metabolites.

The stress tolerance in the isolated bacterial microflora could be autonomous entities to facilitate crop growth under exceptional and marginal environments. The attributes of endophytic microflora in this context have been reported previously^63^. The inoculation experiments under pot house conditions revealed that all the bacterial strains caused a considerable increase in the host plant's growth and yield. The germination percentage, root, and shoot dry weights increased significantly in all the inoculations against the control. Similar observations were recorded regarding the product per plant and oil content (%) in all the bacterial inoculations. The disease intensity was as low as 10.44% in the SKUAST-K46 inoculated strain against the control (48.09%). Similar to our results, Dawwan et al.^64^ reported significant improvements in vegetative growth parameters of inoculated plants as compared to uninoculated control. Shi et al*.*^65^ also found a substantial increase in fresh and dry weight plant height when inoculated beetroots with selected endophytic bacterial isolates. Padder et al*.*^66^ reported an increase in germination percentage up to 95%, disease incidence containment [17% against the control (76.09%)], shoot length, and root length as high as 25–50% in *Dalbergia sissoo* inoculated with bacterial isolates due to their ability to produce a plethora of antifungal compounds like chitinase. The reduced disease incidence and intensity and stress alleviation have been the prominent attribute of bacterial endophytic formulations due to their ability to enhance the different gene expressions coupled with elevated biochemical response in the host^1^. The present investigation's increased germination percentage, yield, and growth could inhibit some prevalent seed-associated phytopathogens by cascading bacteria-associated metabolites. Chitinase seems to play a central role in phytopathogen containment^66^. This could be why the highest chitinase-producing strain smmppsap2 resulted in the highest germination percentage, ultimately alleviating the host's biotic stress and ultimately good yield and increased plant growth. Similar findings were reported by padder et al.^67–69^ with *Pseudomonas fluorescence* strain smmppsap5.

## Conclusion

This study offers acumens on the endophytic microbiome of *B. rapa*, which has not been characterized previously. The conclusions of the present investigation could be summarized as (a) *B. rapa* plant sustains a diverse endophytic microbiome which varies mainly between the sampling sites across the Himalayan germplasm. (b) The chitinase-producing ability in the sampled germplasm reflected dynamism in the endophytic community. (c) The presence of plant growth-promoting traits and chitinase production in the isolated culturable microfloral strains suggests that there is the possibility that these endophytes contribute to the growth, development and disease standing of the crop. (e) The stress tolerance attributes in the isolated microfloral strains suggest that these candidates can be instrumental in helping the crop to sustain the changing climate-mediated stress, both temperature and drought. (f) The controlled experiment studies reflect that there is the possibility that these strains can be applied as bioinoculant formulations at the field level. These concepts suggest that plants and endophyes evolve together within a particular niche. The various factors shape the endophytic symbiosome; these factors need to be studied more extensively to decisively determine the fundamental elements that determine the dynamics of endophytic microflora across the germplasm of *B. rapa*.

## Materials and methods

“For the isolation of endophytic bacterial isolates, the roots of *B. rapa* were collected from; Anantnag, Srinagar, and Baramulla, J&K India (33° 49′ 00. 09″ N 75° 15′ 33. 01″ E to 34° 00′ 32. 20″ N 74° 47′ 12. 83″ E).

### Isolation

The surface sterilization technique was employed with some modifications^15^. Before separating the endophytes, the collected samples were washed under tap water, then dried by soaking in 70% ethanol for one minute. These dried samples were then treated with three percent sodium hypochlorite for 30 s. Lastly, subjected to washing with sterile distilled water (thrice) and then dried using tissue paper (sterilized). The samples, after getting through the process of surface disinfection, were placed for grinding with a sterile mortar pestle, and then the ground samples were dissolved in phosphate buffer saline (1000 ml containing 1.44 g Na_2_HPO_4_; 2.4 g KH_2_HPO_4_; 0.2 g KCl; 8.0 g NaCl and a pH of 7.4). Ground plant material was then serially diluted, and a volume of 100 µl from apiece dilution of 10^–2^, 10^–3^, 10^–6^, 10^–9^ were spread plated on solid growth media (trypticase soy agar) followed by incubation at 37 °C for 24–72 h. The sterilizers' (sodium hypochlorite) surface sterilization performance was tested by inoculating surface sterilizing the whole root on tryptic soy/agar (TSA; Merck Co., Germany) media plates; the no growth indicated surface sterilization.

The morphological appearances, such as shape, color, and growth features of all the colonies, were examined. Dissimilar bacterial colonies were selected and sub-cultured. Each bacterial endophytic culture was assigned a specific code. All the isolated cultures were preserved at − 80 °C in a solution of 20% glycerol for further studies.

### Diversity analysis and molecular description with 16S rRNA and ARDRA analysis

The CTAB procedure (N-Cetyl-N, N-trimethyl-ammonium bromide) was used to isolate the total genetic Material^70–72^. For 16S rDNA amplification, both forward and reverse primers viz., fD1 (5′ AGAGTTTGATCCTGGCTCAG 3′) and rD1 (5′ AAGGAGGTGATCCAGCCGCA 3′)^73^ were used in a thermocycler (BioRad, USA). Amplification materials were resolved on a 1.5 percent agarose gel and interpreted using a gel doc framework (Alfa Imager, Alfa Innotech Corporation, USA). Genie PureTM fast PCR purification kit (GeNeiTM, Bengaluru, India) was used to purify the amplicons, then quantified at 260 nm using a spectrophotometer using calf thymus DNA as a control. Restriction fragment length polymorphism of amplified 16S ribosomal DNA with RsaI and HhaI (Thermo scientific) employed the method^1^ to avoid duplicity among the isolated bacterial strains. In an Applicable Biosystems 3130 Genomic Analysis tool, the purified partial 16S rDNA amplification were sequenced (Applied Biosystems, CA, and the USA).

### Analysis of 16S rDNA sequences

The Nucleotide Basic Local Search Tool (BLAST N) Program (www.ncbi.nlm.nih.gov/ BLAST)' was used to compare the partial nucleotide sequences with already existing arrangements in the NCBI database, the lines with > 99% similarity were retrieved. All the deals were trimmed for the Neighbor-Joining Tree construction, engaging trim primers for mapped reads (the reads hitting the primer parameters using primer track) in the CLC package (QUAGEN Bioinformatics). All the sequences were deposited in NCBI at https://submit.ncbi.nlm.nih.gov/about/genbank. The datasets generated in the current investigation are available in NCBI at https://www.ncbi.nlm.nih.gov with accession numers (Table S2). Phylogenetic analysis and multiple cluster alignment were carried out using the MEGA package (v.10.2.6) founded on the neighbor-joining method using a one-kilo repetition bootstrap. The diversity analysis of the isolated taxonomic entities was estimated employing Past 3 software (v 4.03) and EstimateS (v.9.1.1).

### Chitinase activity

To screen the isolated bacterial microflora for chitinase activity, the bacterial strains after serial dilution were streak plated on (15μL of 3 × 10^9^ CFU/mL) fresh medium [2.0 g colloidal chitin (crustacean); 0.1 g KH_2_PO_4_; 0.01 g MgSO_4_.7H_2_O; 3.0 g NaCl; 0.7 g (NH_4_)_2_SO_4_; 0.05 g yeast extract; 2.0 g agar and 50 mM of sodium phosphate buffer, pH 6.0 (all the components are given on per liter basis)] at 30 °C over three days, after incubation, the bacterial strains were examined for the development of clearance zone on the solid media, and its presence indicated the presence of chitinase activity. The clearance zone and colony diameter measurements were made to calculate the CS: CZ ratio. To determine chitinase activity (U/mL), Berger and Reynold's method calculated chitinase activity^74^. The same media (100 mL) except the agar was taken in 250 ml Erlenmeyer flasks and inoculated with each chitinase positive bacterial strain (15μL of 3 × 10^9^ CFU/mL log-phase cells) and incubated at 30 °C for 72 (hrs.). After incubation, the enzyme mixture was collected by centrifuging the mixture for 20 min at 12,000 rpm at room temperature. The substrate and enzyme mixture [0.5 ml (1% w/v of colloidal chitin in minimal media) and 0.5 ml of enzyme solution] was incubated at 45 °C for 1 h. The reaction was then stopped by 3 ml of a 5-dinitrosalicylic acid reagent and then 'heated for five minutes at 100 °C to cease the reaction completely.' The supernatant (collected by centrifuging the heated mixture at 10,000 rpm for 15 min at 4 °C) was collected and subjected to absorbance measurement with a UV (530 nm) spectrometer. For determination of enzyme unit, serial dilutions of Nacetylglosamine (from 0 to 50 mM) were prepared. One unit (U) of the chitinase activity was defined as the amount of enzyme required to release one mmol of N-acetyl D glucosamine (as a standard) from chitin/min. The experimental protocol was modified as per the desired set of conditions to carry out experiments on the effect of various carbohydrate sources and different incubation periods.

### Biochemical characterization of bacterial isolates

All chitinase-producing endophytic bacterial isolates were characterized based on biochemical features as per Bergey's manual of determinative Bacteriology^75^.

### Indole-3-acetic acid (IAA) production

The Salkowski method of color development was employed for calculating IAA^76^."The bacterial endophytic strains were inoculated into 25 mL nutrient broth fortified with 0.2 mg/mL L-tryptophan and incubated in a BOD incubator at 28 °C. Following incubation of 72 h, 2 mL per cultivation broth was centrifuged for two minutes at 7,000 rpm, followed by the addition of the same amount of Salkowski's reagent (1 mL 0.05 M FeCl_3_ in 50 mL 35 percent HClO_4_) to cultivation supernatant for the colored complex production which was estimated calorimetrically at 500 nm wavelength using a spectrophotometer.

### Phosphate solubilization

The TSA broth-developed bacterial strains were placed [3 × 10^9^ CFU/mL log phase developing cells (15μL)] on Pikovskaya's medium plates for the detection of solubilization zone^77^. These plates were incubated for 4 days at 28 °C. The established solubility region and colony diameter measured were used to calculate the solubilization index (SI = diameter of the colony/ diameter of halozone/colony diameter)^78^. Bray and Kartz were employed using Pikovskaya's broth^79^. The cultures inoculated in Pikovskaya's broth [3 × 10^9^ CFU/mL log phase developing cells (15μL)] were incubated for 4 days at 28 °C. The soluble phosphorus released in the broth was estimated calorimetrically.

### Production of ammonia and hydrocyanic acid

The log phase developing cells (15μL of [3 × 10^9^ CFU/mL] were inoculated in peptone water (10 mL) and incubated for (72 h at 30 °C). Approximately (0.5 mL) Nessler reagent was added, and the ammonia output's yellow/brown pigment appearance was recorded^80^. The culture was inoculated after incubation was centrifuged for 15 min at 10,000 rpm for quantitative estimation. To the collected supernatant (1 mL), a volume of 1 mL of the Nessler reagent was added. To this mixture, purified water was added for serial dilution because of the sensitivity of the calculation method to higher ammonia concentrations. Brown/yellow color was developed, and absorbance was measured at 630 nm’. The ammonia volume was determined by the ammonium sulfate level (0.1–1 mol/mL) curve^81^.

The King's B medium was mixed with 4.4 g/L of glycine to measure the hydrocyanic acid (HCN)^82,83^. ‘The degree of color shift from yellow to dark brown on filter paper dipped into 2% sodium carbonate (prepared in picric acid) was determined. The bacteria (15μL [3 × 10^9^ CFU/mL were grown in the glycine-modified King's B broth, and the regular filter paper strip (100 × 15 mm^2^) in alkaline picrates was soaked and hanged for quantitative testing in the conical flask^84^. After the filter paper was inserted into a test tube containing 10 mL of filtered water, the absorption of this colored water was estimated at 625 nm.

### Siderophore estimation

The liquid Chrome Azurol S (CAS) assay was used for siderophore estimation^85^. A 0.5 mL of Chrome Azurol-S (CAS) was added to 0.5 mL of supernatant (1 mL) of the cell-free extract [obtained by inoculating 15μL of 3 × 10^9^ CFU/mL log-phase cells into the nutrient broth and after incubation was centrifuged for 15 min at 10,000 rpm] besides 10 µL shuttle solution (0.2 M 5-Sulfosalicylic acid). The absorption range was estimated at 630 nm after 10 min of incubation at room temperature. The media (minimal) was taken as the reference (made with the same ingredients as the minimal medium, except for the addition of cell-free culture-supernatant extract). Siderophore units were obtained from the following equation:

$$\left[ {{\text{Percent}}\;{\text{Siderophore}}\;{\text{Unit}} = \left( {{\text{Ar}} - {\text{As}}/{\text{As }} = { 1}00} \right)} \right].$$

where [Ar is the reference absorption at (630 nm)].

[As is the (630 nm) testing absorbance].

### Antifungal activity

All of the chitinase-producing bacterial strains were tested against 6 phytopathogenic strains :(*Fusarium oxysporum, Fusarium solani, Pythium amphidermatum, Dematophora necatrix*^*1*^*, Rhizoctonia solani, and Dematophora necatrix*^*2*^*).* The bacterial endophytes (48 h. old culture) full-grown on nutrient agar Petri dishes were placed (as line streaks) on PDA at an equal distance of 2.2 cm from the center of the 100 × 15 mm Petri dish. A 5 mm disc taken from the 10-day old fungal culture was placed at the center of the Petri dish as per the protocol^86^ with some modifications. The plates were incubated at 23 °C. The plates without the bacterial endophyte were kept under control. The experiment was repeated per the required replications set for the experimental design.

*‘*The formula calculated the percentage growth inhibition:

$${\text{Control}} - {\text{Treatment}}/{\text{Control }} \times {1}00$$

### Antibiotic sensitivity

Antibiotic susceptibility testing was carried out using discs impregnated with antibiotics (6 mm diameter). The strains' antibiotic susceptibility was assessed using the Kirby Bauer disc-diffusion assay system against chloramphenicol, rifampicin, amikacin, erythromycin, and polymyxin-B (analytical grade)^87^. ‘Antibiotics were used in amounts of 35.0 µg per disk. According to the DIFCO Manual 10th edition, strains were classified as resistant or responsive based on the inhibition zone reported^88^.

### Stress tolerance

The bacterial isolates were grown at different PEG concentrations (10, 15, 20, and 25%) and at different temperature ranges (15, 25, and 35 °C) in nutrient broth as per the local climatic conditions to observe their ability to grow in possible extreme conditions of the zone; the CFU/mL was measured.

Formulation Development.

### In vivo* studies*

The effect of isolated cultures on the growth of *B. rapa* was ascertained under pot culture conditions by using it as a host plant (*B. rapa*). Seeds of *B. rapa* were surface sterilized by 0.2% sodium hypochlorite. They were sown in pots of 4 kg capacity containing 2 kg silty clay loam soil and inoculated with individual isolates. All the pots were inoculated with 3 ml inoculum of log-phase bacterial isolates every 30 days till the harvesting of the crop. A control was also maintained by inoculating with broth devoid of bacterial culture. The experiment was conducted in a completely randomized design, with each treatment replicated four times. The pots were irrigated as and when needed. At the crop's maturity, the plants were uprooted, and observations were recorded for various growth and yield attributes, viz*.,* oil content, yield per plant, dry root weight, and shoot dry weight. The yield per plant was calculated by collecting the seeds per plant and weighing them. The germination percentage was calculated as per the method adopted by^89^. Oil content was determined by following the solvent extraction technique^90^, for which 3 g of *B. rapa* seeds were first crushed in 3 g Na_2_SO_4,_ and the resultant powder containing oil was taken into test tubes; then 20 ml hexane was poured into the test tubes as mobile phase. Elute-containing oil was stored in a vile, and hexane was evaporated in a hot water bath. The remaining oil was weighed, and its percentage was calculated using the following formulae:

$${\text{Oil}}\;{\text{percentage}} = \frac{{{\text{Oil}}\;{\text{content}}}}{{{\text{Seed}}\;{\text{weight}}}} \times {1}00$$

#### Measurement of disease intensity

The *Sclerotinia sclerotiorum* infected plant samples of *B. rapa* were collected from the plains of J&K, and the cultures were introduced in the plants grown under controlled conditions; the disease incidence was recorded every three weeks by counting the number of infected leaves, and visually estimating their per-centage diseased area. Observations were made for five randomized branches per plant, and ten older leaves were evaluated to assess the disease incidence and severity using the following formula:

$$\% {\text{Disease}}\;{\text{incidence}} = {\text{number}}\;{\text{of}}\;{\text{infected}}\;{\text{leaves}}/{\text{total}}\;{\text{number}}\;{\text{of}}\;{\text{tested}}\;{\text{leaves}} \times {1}00$$

The experiment was carried out in a completely randomized design.

### Approval for plant experiments

Confirm that all experiments were performed following relevant guidelines and regulations. Also, permission was taken for the collection of plant specimens.

## Statistical analysis

The experiment was conducted in a randomized block configuration. The results were analyzed at a significance level of 0.05; the mean values were compared from five observations/replications in IBM SPSS Statistics 19.0 (SPSS, Inc., Chicago, IL, USA). The cluster analysis and diversity indices were calculated using Past 3 software (v 4.03) and EstimateS (v.9.1.1).

## Supplementary Information

Supplementary Information.

## References

[1] Padder SA, Mansoor S, Bhat SA, Baba TR, Rather RA, Wani SM, Popescu SM, Sofi S, Aziz MA, Hefft DI (2021). Bacterial endophyte community dynamics in apple (*Malus**domestica* Borkh.) germplasm and their evaluation for scab management strategies. J. Fungi.

[2] Wei J, Chen F, Liu Y, Abudoukerimu A, Zheng Q, Zhang X, Sun Y, Yimiti D (2020). Comparative metabolomics revealed the potential antitumor characteristics of four endophytic fungi of *Brassica rapa* L. ACS Omega.

[3] Shahzad R, Khan AL, Bilal S, Lee IJ (2018). What is there in seeds? Vertically transmitted endophytic resources for sustainable improvement in plant. Growth Front Plant Sci..

[4] Afzal I, Shinwari ZK, Sikandar S, Shahzad S (2019). Plant beneficial endophytic bacteria: Mechanisms, diversity, host range and genetic determinants. Microbiol. Res..

[5] Emami S, Alikhani HA, Pourbabaei AA, Etesami H, Sarmadian F, Motessharezadeh B (2019). Effect of rhizospheric and endophytic bacteria with multiple plant growth promoting traits on wheat growth. Environ. Sci. Pollut. Res..

[6] Kumar V, Jain L, Jain SK, Chaturvedi S, Kaushal P (2020). Bacterial endophytes of rice (Oryza sativa L.) and their potential for plant growth promotion and antagonistic activities. South Afr. J. Bot..

[7] Rana KL, Kour D, Kaur T, Devi R, Yadav AN, Yadav N, Dhaliwal HS, Saxena AK (2020). Endophytic microbes: Biodiversity, plant growth-promoting mechanisms and potential applications for agricultural sustainability. Antonie Van Leeuwenhoek.

[8] Hazarika SN, Saikia K, Borah A, Thakur D (2021). Prospecting endophytic bacteria endowed with plant growth promoting potential isolated from camellia sinensis. Front. Microbiol..

[9] Etminani F, Harighi B (2018). Isolation and identification of endophytic bacteria with plant growth promoting activity and biocontrol potential from wild pistachio trees. Plant Pathol. J..

[10] Pascale A, Proietti S, Pantelides IS, Stringlis IA (2020). Modulation of the root microbiome by plant molecules: The basis for targeted disease suppression and plant growth promotion. Front. Plant Sci..

[11] Sangwan P, Raj K, Wati L, Kumar A (2021). Isolation and evaluation of bacterial endophytes against Sclerospora graminicola (Sacc.) Schroet, the causal of pearl millet downy mildew. Egypt. J. Biol. Pest Control..

[12] Trivedi P, Leach JE, Tringe SG, Sa T, Singh BK (2020). Plant–microbiome interactions: From community assembly to plant health. Nat. Rev. Microbiol..

[13] Restu M, Bachtiar B, Larekeng SH (2020). Gibberellin and IAA production by rhizobacteria from various private forest. IOP Conf. Ser.: Earth Environ. Sci..

[14] Leroy N, de Tombeur F, Walgraffe Y, Cornélis JT, Verheggen FJ (2019). Silicon and plant natural defenses against insect pests: Impact on plant volatile organic compounds and cascade effects on multitrophic interactions. Plants..

[15] Mehdizadeh, M. & Mushtaq, W. Biological control of weeds by allelopathic compounds from different plants: A bioherbicide approach. In *Natural Remedies for Pest, Disease and Weed Control*, 107–117 (Academic Press, 2020).

[16] Wang S, Liang H, Liu L, Jiang X, Wu S, Gao H (2020). Promiscuous enzymes cause biosynthesis of diverse siderophores in Shewanella oneidensis. Appl. Environ. Microbiol..

[17] Benidire L, Madline A, Pereira SIA, Castro PML, Boularbah A (2021). Synergistic effect of organo-mineral amendments and plant growth-promoting rhizobacteria (PGPR) on the establishment of vegetation cover and amelioration of mine tailings. Chemosphere.

[18] Abdel Latef AAH, Omer AM, Badawy AA, Osman MS, Ragaey MM (2021). Strategy of salt tolerance and interactive impact of azotobacter chroococcum and/or alcaligenes faecalis inoculation on canola (*Brassica**napus* L.) plants grown in saline soil. Plants..

[19] Atanasov AG, Zotchev SB, Dirsch VM, Supuran CT (2021). Natural products in drug discovery: Advances and opportunities. Nat. Rev. Drug Discov..

[20] Mohiddin FA, Padder SA, Bhat AH, Ahanger MA, Shikari AB, Wani SH, Bhat FA, Nabi SU, Hamid A, Bhat NA, Sofi NR (2021). Phylogeny and optimization of trichoderma harzianum for chitinase production: Evaluation of their antifungal behaviour against the prominent soil borne phyto-pathogens of temperate India. Microorganisms.

[21] Sheikh HMA, Hamshary OI, Abd El-Hafez AEN (2022). Molecular identification, characterization and improvement of a chitinase producing bacillus strain showing significant control against some dermatophytic fungi. J. Pure Appl. Microbiol..

[22] Oyeleye A, Norm YM (2018). Chitinase: Diversity, limitations, and trends in engineering for suitable applications. Biosci. Rep..

[23] Xie S, Feng H, Yang F, Zhao Z, Hu X, Wei C (2019). Does dual reduction in chemical fertilizer and pesticides improve nutrient loss and tea yield and quality? A pilot study in a green tea garden in Shaoxing, Zhejiang Province, China. Environ. Sci. Pollut. Res..

[24] Santoyo G, Moreno-hagelsieb G, Orozco-mosqueda C, Glick BR (2016). Plant growth-promoting bacterial endophytes. Microbiol. Res..

[25] Liu FP, Liu HQ, Zhou HL, Dong ZG, Bai XH, Bai P (2014). Isolation and characterization of phosphate-solubilizing bacteria from betel nut (*Areca catechu*) and their effects on plant growth and phosphorus mobilization in tropical soils. Biol. Fertil. Soils.

[26] Rakow G, Pua EC, Douglas CJ (2004). Species origin and economic importance of brassica. Brassica Biotechnology in Agriculture and Forestry.

[27] Johnson JS, Spakowicz DJ, Hong BY, Petersen LM, Demkowicz P, Chen L (2019). Evaluation of 16S rRNA gene sequencing for species and strain level microbiome analysis. Nat. Commun..

[28] Segaran G, Sathiavelu M (2019). Fungal endophytes: A potent biocontrol agent and a bioactive metabolites reservoir. Biocatal. Agric. Biotechnol..

[29] Verma, P. P., Shelake, R. M., Das, S., Sharma, P. & Kim, J. Y. Plant growth-promoting rhizobacteria (PGPR) and fungi (PGPF): Potential biological control agents of diseases and pests. In *Microbial Interventions in Agriculture and Environment*, 281–311 (Springer, 2019).

[30] Wang X, Xiao C, Ji C, Liu Z, Song X, Liu Y, Li C, Yan D, Li H, Qin Y, Liu X (2021). Isolation and characterization of endophytic bacteria for controlling root rot disease of *Chinese jujube*. J. Appl. Microbiol..

[31] Passari AK, Mishra VK, Saikia R, Gupta VK, Singh BP (2015). Isolation, abundance and phylogenetic affiliation of endophytic actinomycetes associated with medicinal plants and screening for their in vitro antimicrobial biosynthetic potential. Front. Microbiol..

[32] Pisarska K, Pietr S (2015). Biodiversity of dominant cultivable endophytic bacteria inhabiting tissyes of six different cultivars of Maize (*Zea**mays* L. ssp mays). Pol. J. Microbiol..

[33] Jimenez-Gomez A, Saati-Santamaría Z, Kostovcik M, Rivas R, Velazquez E, Mateos PF, Menéndez E, Garcca-Fraile P (2020). Selection of the root endophyte Pseudomonas brassicacearum CDVBN10 as plant growth promoter for Brassica napus L. crops. Agronomy.

[34] Arnold AE, Lutzoni F (2007). Diversity and host range of foliar fungal endophytes: Are tropical leaves biodiversity hotspots?. Ecology.

[35] Garcia K, Zimmermann SD (2014). The role of mycorrhizal associations in plant potassium nutrition. Front. Plant Sci..

[36] Basumatary B, Das D, Choudhury BN, Dutta P, Bhattacharyya A (2021). Isolation and characterization of endophytic bacteria from tomato foliage and their in vitro efficacy against root-knot nematodes. J. Nematol..

[37] Papik J, Folkmanova M, Polivkova-Majorova M, Suman J, Uhlik O (2020). The invisible life inside plants: Deciphering the riddles of endophytic bacterial diversity. Biotechnol. Adv..

[38] Trivedi P, Mattupalli C, Eversole K, Leach JE (2021). Enabling sustainable agriculture through understanding and enhancement of microbiomes. New Phytol..

[39] Mekasha S, Tuveng TR, Askarian F, Choudhary S, Schmidt-Dannert C, Niebisch A, Modregger J, Vaaje-Kolstad G, Eijsink VG (2020). A trimodular bacterial enzyme combining hydrolytic activity with oxidative glycosidic bond cleavage efficiently degrades chitin: A chitinolytic enzyme with hydrolytic and oxidative activity. J. Biol. Chem..

[40] Gomaa EZ (2021). Microbial chitinases: Properties, enhancement and potential applications. Protoplasma.

[41] Dole NP, Dar MA, Pandit RS (2021). Recent advances in the bioprospection and applications of chitinolytic bacteria for valorisation of waste chitin. Arch. Microbiol..

[42] Benito Santano, P. Comparative proteomic and transcriptomic profiling of *Micromonospora* strains associated with legumes. In *Universidad de Salamanca*, 1 (2020).

[43] Banerjee S, Mukherjee A, Dutta D, Ghosh K (2021). Evaluation of chitinolytic gut microbiota in some carps and optimization of culture conditions for chitinase production by the selected bacteria. J. Microbiol. Biotechnol. Food Sci..

[44] Dukariya G, Kumar A (2021). Statistical optimization of chitinase production by Box-Behnken design in submerged fermentation using Bacillus cereus GS02. J. Appl. Biol. Biotechnol..

[45] Dey R, Raghuwanshi R (2020). Comprehensive assessment of growth parameters for screening endophytic bacterial strains in *Solanum lycopersicum* (Tomato). Heliyon..

[46] Mishra I, Fatima T, Egamberdieva D, Arora NK (2020). Novel bioformulations developed from pseudomonas putida BSP9 and its biosurfactant for growth promotion of *Brassica**juncea* (L.). Plants..

[47] Adhikari P, Jain R, Sharma A, Pandey A (2021). Plant growth promotion at low temperature by phosphate-solubilizing pseudomonas Spp. isolated from high-altitude himalayan soil. Microbial. Ecol..

[48] Lipková N, Medo J, Artimová R, Maková J, Petrová J, Javoreková S, Michalko J (2021). Growth promotion of rapeseed (Brassica napus L.) and blackleg disease (Leptosphaeria maculans) suppression mediated by endophytic bacteria. Agronomy.

[49] Sundaram, L., Rajendran, S. & Subramanian, N. Metal Stress Impacting Plant Growth in Contaminated Soil Is Alleviated by Microbial Siderophores. *Role of Microbial. Commun. Sustain*. 317–332 (2021).

[50] Wang JY, Guo C, Zhao P, Yu FY, Su Y, Qu JP, Wang JL, Lin RS, Wang B, Gao Z, Yang ZY (2021). Biocontrol potential of Bacillus altitudinis AMCC1040 against root-knot nematode disease of ginger and its impact on rhizosphere microbial community. Biol. Control.

[51] Adhikari P, Pandey A (2020). Bioprospecting plant growth promoting endophytic bacteria isolated from Himalayan yew (*Taxus**wallichiana* Zucc.). Microbiol. Res..

[52] Pavithra G, Bindal S, Rana M, Srivastava S (2020). Role of endophytic microbes against plant pathogens: A review. Asian J. Plant Sci..

[53] Azizoglu U, Yilmaz N, Simsek O, Ibal JC, Tagele SB, Shin JH (2021). The fate of plant growth-promoting rhizobacteria in soilless agriculture: Future perspectives. 3 Biotech.

[54] Shakeela S, Padder SA, Bhat ZA (2017). Isolation and characterization of plant growth promoting rhizobacteria associated with medicinal plant Picrorhiza Kurroa. J. Pharmacognosy Phytochemistr..

[55] Hayat S, Hasan SA, Hayat Q, Ahmad A (2010). Brassinosteroids protect Lycopersicon esculentum from cadmium toxicity applied as shotgun approach. Protoplasma.

[56] Singh K, Rani A, Padder SA, Gera R (2017). Plant Growth Promoting (PGP) Attributes of stress tolerant Rhizobial isolates from root nodules of Pigeon pea [Cajanus cajan (L.) Millspaugh] growing in Haryana, India. Int. J. Curr. Microbiol. Appl. Sci..

[57] Sivasakthi S, Usharani G, Saranraj P (2014). Biocontrol potentiality of plant growth promoting bacteria (PGPR)-Pseudomonas fluorescens and Bacillus subtilis: A review. Afr. J. Agric. Res..

[58] Mishra, J., Fatima, T. and Arora, N.K., Role of secondary metabolites from plant growth-promoting rhizobacteria in combating salinity stress. In *Plant Microbiome: Stress Response*, 127–163 (Springer, 2018).

[59] Sharma, N., Manhas, R. K., Bhardwaj, R. & Ohri, P. Bioefficacy of bio-metabolites produced by streptomyces sp. strain MR-14 in ameliorating meloidogyne incognita stress in solanum lycopersicum seedlings. *J. Plant Growth Regul.*,1–13.( 2021)

[60] Su Z, Cai S, Liu J (2021). Root-associated endophytic bacterial community composition of asparagus officinalis of three different varieties. Indian J. Microbiol..

[61] de Menezes AB, Richardson AE, Thrall PH (2017). Linking fungal–bacterial co-occurrences to soil ecosystem function. Curr. Opin. Microbiol..

[62] Padder SA, Dar GH, Bhat ZA, Verma K, BashirWani A (2017). Morphological metabolic and biochemical characterization of bacterial root endophytes associated with brown sarson (*Brassica**rapa* L.). J. Pharmacognosy Phytochemistry..

[63] Dawwam GE, Elbeltagy A, Emara MH, Abbas HI, Hassan MM (2013). Beneficial effect of plant growth promoting bacteria isolated from the roots of potato plant. Ann. Agric. Sci..

[64] Shi Y, Lou K, Li C (2009). Promotion of plant growth by phytohormone-producing endophytic microbes of sugar beet. Biol. Fertilizers Soils..

[65] Padder SA, Bhat ZA, Sofi S, Mukhtar M (2015). Biochemical attributes of efficient PGPR bioinoculants and their effect on growth of dalbergia sissoo (Roxb.). J. Pure Appl. Microbiol..

[66] Rather RA, Bano H, Perveen K, Bukhari NA, Padder SA, Baba TR, Qureshi A, Khan NA, Khan AH, Samaraweera H (2022). Antifungal potential of Colchicum luteum and determination of colchicine content using HPLC for application as a fungicide. J. King Saud Univ.-Sci..

[67] Padder SA, Dar GH, Mohiddin FA, Shah MD (2016). Characterization and plant growth promoting aspects of a novel phosphate solubilizing brown sarson endophyte pseudomonas fluorescens strain smppsap5 isolated from Northern Himalayas of India. J. Pure Appl. Microbiol..

[68] Crosbie DB, Mahmoudi M, Radl V, Brachmann A, Schloter M, Kemen E, Marín M (2022). Microbiome profiling reveals that Pseudomonas antagonises parasitic nodule colonisation of cheater rhizobia in Lotus. New Phytol..

[69] Huang S, Chen X, Yan R, Huang M, Chen D (2022). Isolation, identification and antibacterial mechanism of the main antibacterial component from pickled and dried mustard (Brassica juncea Coss. Var. foliosa Bailey). Molecules.

[70] Doyle JJ, Doyle JL (1987). A rapid DNA isolation procedure for small quantities of fresh leaf tissue. Phytochem. Bull..

[71] Doyle JJ, Dickson EE (1987). Preservation of plant samples for DNA restriction endonuclease analysis. Taxon.

[72] Culings KW (1992). Design and testing of a plant specific PCR primer for ecological and evolutionary studies. Mol. Ecol..

[73] Lukow T, Dunfield PF, Liesack W (2000). Use of the t-RFLP technique to assess spatial and temporal changes in the bacterial community structure within an agricultural soil planted with transgenic and non-transgenic potato plants. FEMS Microbial. Ecol..

[74] Berger LR, Reynold DM (1958). The chitinase system of a strain of Streptomyces griseus. Biochem. Biophys. Acta..

[75] Holt, J. G.*The shorter Bergey's manual of determinative bacteriology*. The shorter Bergey's manual of determinative bacteriology. 8th edition (1977).

[76] Tang YW, Bonner J (1974). The enzymatic inactivation of IAA. Some characteristics of the enzyme contained in pea seedlings. Arch. Biochem..

[77] Pikovskaya RI (1948). Mobilization of phosphorus in soil in connection with vital activity of some microbial species. Mikrobiologiya.

[78] Premono, M. E., Moawad, A. M. & Vlek, P. L. G. Effect of phosphate-solubilizing Pseudomonas putida on the growth of maize and its survival in the rhizosphere (No. REP-12113. CIMMYT.) (1996).

[79] Bray RH, Kurtz LT (1945). Determination of total, organic, and available forms of phosphorus in soils. Soil Sci..

[80] Cappuccino JG, Sherman N (1999). Microbiology-A Laboratory Manual.

[81] Demutskaya LN, Kalinichenko IE (2010). Photometric determination of ammonium nitrogen with the nessler reagent in drinking water after its chlorination. J. Water Chem. Tech..

[82] King EO, Ward MK, Raney DE (1954). Two simple media for the demonstration of Pyocyanin and fluorescin. J. Lab. Clin. Med..

[83] Baker PD, Schippers BOB (1987). Microbial cyanide production in the rhizosphere in relation to potato yield production and Pseudomonas spp. mediated plant growth stimulation. Soil Biol. Biochem..

[84] Sadasivam S, Manickam A (1992). Biochemical Methods for Agricultural Sciences.

[85] Schwyn B, Neilands JB (1987). Universal chemical assay for the detection and determination of siderophores. Anal. Biochem..

[86] Kumar V, Kumar A, Pandey KD, Roy BK (2015). Isolation and characterization of bacterial endophytes from the roots of Cassia tora L. Ann. Microbiol..

[87] Bauer AW, Perry DM, Kirby WM (1959). Single-disk antibiotic-sensitivity testing of staphylococci: An analysis of technique and results. A.M.A. Arch. Intern. Med..

[88] Difco. Difco manual, 10th ed. Difco Laboratories, Detroit, MI. (1984).

[89] Joshi S, Jaggi V, Tiwari S, Sah VK, Sahgal M (2019). Multitrate phosphate solubilizing bacteria from Dalbergia sissoo Roxb. Rhizosphere in natural forests of Indian Central Himalayas. Environ. Ecol..

[90] Irfan M, Ahmad A, Hayat S (2014). Effect of cadmium on the growth and antioxidant enzymes in two varieties of Brassica juncea. Saudi J. Biol. Sci..

