# Supplementary material for: Dynamics, phylogeny and phyto-stimulating potential of chitinase synthesizing bacterial root endosymbiosiome of North Western Himalayan Brassica rapa L
Source: Sci Rep. 2022 Apr 25;12:6742. doi: 10.1038/s41598-022-11030-0 (PMC9038727; doi:10.1038/s41598-022-11030-0)
Supplement: Supplementary file 1 — Supplementary Information. [file 41598_2022_11030_MOESM1_ESM.docx]

**Supplementary Material**

Shahid A. Padder^1*^, Rauoof Ahmad Rather^2*^, Sajad Ahmad Bhat^1^, M. D. Shah^3^, Tawseef

Rehman Baba^4^, N M Mubarak^5*^

^1^Division of Basic Sciences and Humanities, FoH, Sher-e-Kashmir University of Agricultural Sciences & Technology of Kashmir, Srinagar 190025, Kashmir, J&K, India.

^2^Division of Environmental Sciences,FoH, Sher-e-Kashmir University of Agricultural Sciences & Technology of Kashmir 190025, Kashmir, Srinagar, J&K, India.

^3^Division of Plant Pathology, FoH, Sher-e-Kashmir University of Agricultural Sciences & Technology of Kashmir, Srinagar 190025, Kashmir, J&K, India.

^4^Division of Fruit Science, FoH, Sher-e-Kashmir University of Agricultural Sciences & Technology of Kashmir, Srinagar 190025, Kashmir, J&K, India.

^5^Petroleum and Chemical Engineering, Faculty of Engineering, Universiti Teknologi Brunei, Bandar Seri Begawan BE1410, Brunei Darussalam

*Correspondence: Shahid Ahmad Padder (S.A.P); [shahidpadder@skuastkashmir.ac.in](mailto:shahidpadder@skuastkashmir.ac.in), [rouf.haq@gmail.com](mailto:rouf.haq@gmail.com); mubarak.yaseen@gmail.com

**Table (S1): Details of the sites from which Brown Sarson (*Brassica rapa* L.) germplasm was collected**

| **S. No.** | **District** | **Block/ Village** | **Coordinates** | **Isolates obtained** | **Log_10_ ( 10^5^ Cfu/mL)** |
| --- | --- | --- | --- | --- | --- |
| **1.** | **Anantnag** | **Block Dachnipora** |  |  |  |
|  |  | 1. Akura | 33^0^ 46**´** 24. 56**″N**  75^0^ 11**´** 13. 98**″E**  5356 ft | **SB1 SB2 SB3 SB15 SB16** | 5.321 |
|  |  |  | 33^0^ 46**´** 40. 01**″N**  75^0^ 10**´** 50. 11**″E**  5346 ft | **SB8 SB41** | 7.342 |
|  |  |  | 33^0^ 47**´** 03. 89**″N**  75^0^ 11**´** 18. 10**″E**  5385 ft | **SB51 SB9** | 7.767 |
|  |  | 1. Bona Nambal | 33^0^ 46**´** 58. 40**″N**  75^0^ 12**´** 26. 35**″E**  5427 ft | **SB22** | 8.209 |
|  |  |  | 33^0^ 47**´** 18. 38**″N**  75^0^ 12**´** 44. 86**″E**  5456 ft | **SB11 SB23** | 7.693 |
|  |  |  | 33^0^ 47**´** 09. 19**″N**  75^0^ 11**´** 51. 46**″E**  5412ft | **SB14**  **SB33 SB27** | 5.294 |
|  |  | 1. Hugam | 33^0^ 47**´** 55. 37**″N**  75^0^ 11**´** 30. 51**″E**  5432 ft | **SB10 SB30** | 8.386 |
|  |  |  | 33^0^ 47**´** 50. 67**″N**  75^0^ 12**´** 19. 90**″E**  5465ft | **SB32** | 8.302 |
|  |  |  | 33^0^ 47**´** 13. 84**″N**  75^0^ 11**´** 20. 84**″E**  5393 ft | **SB48** | 7.959 |
|  |  | **Block Khoverpora** | | |  |
|  |  | 1. Hutmara | 33^0^ 46**´** 49. 69**″N**  75^0^ 13**´** 50. 95**″E**  5467 ft | **SB19 SB25 SB58**  **SB63** | 6.602 |
|  |  |  | 33^0^ 46**´** 58. 13**″N**  75^0^ 14**´** 01. 75**″E**  5471 ft | **SB39 SB40 SB12** | 7.051 |
|  |  |  | 33^0^ 46**´** 44. 85**″N**  75^0^ 13**´** 37. 38**″E**   1. ft | **SB24 SB73** | 7.226 |
|  |  | 1. Panzmulla | 33^0^ 47**´** 53. 49**″N**  75^0^ 16**´** 46. 72**″E**  5744 ft | **SB26 SB34 SB35** | 5.287 |
|  |  |  | 33^0^ 48**´** 16. 42**″N**  75^0^ 16**´** 41. 28**″E**  5759ft | **SB17 SB18** | 5.839 |
|  |  |  | 33^0^ 47**´** 41. 30**″N**  75^0^ 17**´** 26.67**″E**  6100ft | **SB43** | 5.338 |
|  |  | 1. Rakh Chandipora | 33^0^ 49**´** 00. 09**″N**  75^0^ 15**´** 33. 01**″E**  5658 ft | **SB13** | 6.082 |
|  |  |  | 33^0^ 49**´** 20. 50**″N**  75^0^ 16**´** 33. 56**″E**  5792 ft |  | 7.226 |
|  |  |  | 33^0^ 49**´** 28. 69**″N**  75^0^ 16**´** 36. 37**″E**  5745 ft | **SB42 SB44** | 6.327 |
| **2.** | **Srinagar** | **Block Srinagar** | | |  |
|  |  | 1. Nowgam | 34^0^ 01**´** 21. 54**″N**  74^0^ 50**´** 49. 31**″E**  5199 ft | **SB38** | 4.329 |
|  |  |  | 34^0^ 01**´** 16. 07**″N**  74^0^ 50**´** 43. 09**″E**  5185 ft | **SB50** | 4.234 |
|  |  |  | 34^0^ 01**´** 18. 92**″N**  74^0^ 50**´** 48. 94**″E**  5197 ft | **SB4 SB5** | 4.227 |
|  |  | 1. Rawalpora | 34^0^ 01**´** 35. 56**″N**  74^0^ 47**´** 27. 55**″E**  5207 ft | **SB20 SB21** | 5.329 |
|  |  |  | 34^0^ 01**´** 31. 14**″N**  74^0^ 47**´** 28. 41**″E**  5203 ft |  | 7.331 |
|  |  |  | 34^0^ 01**´** 31. 18**″N**  74^0^ 47**´** 27. 79**″E**  5209 ft |  | 5.045 |
|  |  | 1. Rangreth | 34^0^ 00**´** 32. 20**″N**  74^0^ 47**´** 12. 83**″E**  5213 ft | **SB64** | 6.306 |
|  |  |  | 34^0^ 00**´** 25. 41**″N**  74^0^ 47**´** 16. 63**″E**  5210 ft |  | 6.006 |
|  |  |  | 34^0^ 00**´** 35. 83**″N**  74^0^ 47**´** 08. 95**″E**  5220 ft | **SB7** | 5.949 |
|  |  | 1. Zakura | 34^0^ 09**´** 46. 38**″N**  74^0^ 50**´** 08. 86**″E**  5279 ft | **SB76 SB71** | 6.348 |
|  |  |  | 34^0^ 09**´ 32**. 38**″N**  74^0^ 50**´** 28. 09**″E**  5262 ft |  | 6.302 |
|  |  |  | 34^0^ 09**´** 28. 28**″N**  74^0^ 50**´** 13. 67**″E**  5268 ft |  | 6.062 |
|  |  | 1. Gulab Bagh | 34^0^ 10**´** 46. 99**″N**  74^0^ 49**´** 15. 50**″E**  5270 ft | **SB29** | 7.204 |
|  |  |  | 34^0^ 10**´** 46. 11**″N**  74^0^ 49**´** 15. 18**″E**  5271 ft |  | 7.502 |
|  |  |  | 34^0^ 10**´** 28. 91**″N**  74^0^ 48**´** 48. 83**″E**   1. ft |  | 7.842 |
|  |  | 1. Ahmed Nagar | 34^0^ 10**´** 14. 37**″N**  74^0^ 48**´** 43. **″44E**  5279 ft | **SB66 SB69** | 5.330 |
|  |  |  | 34^0^ 09**´** 50. 89**″N**  74^0^ 48**´** 44. 03**″E**  5266 ft |  | 6.081 |
|  |  |  | 34^0^ 10**´** 15. 78**″N**  74^0^ 48**´** 11. 88**″E**  5196 ft |  | 6.116 |
|  |  | 1. Dhara | 34^0^ 10**´** 54. 81**″N**  74^0^ 55**´** 06. 31**″E**  6276 ft | **SB52 SB53** | 5.348 |
|  |  |  | 34^0^ 10**´** 45. 27**″N**  74^0^ 55**´** 14. 29**″E**  6351 ft |  | 5.392 |
|  |  |  | 34^0^ 10**´** 49. 81**″N**  74^0^ 54**´** 17. 73**″E**  6053 ft |  | 5.224 |
|  |  | 1. Tailbal | 34^0^ 09**´** 52. 58**″N**  74^0^ 51**´** 41. 40**″E**  5205 ft | **SB36** | 5.906 |
|  |  |  | 34^0^ 09**´** 55. 85**″N**  74^0^ 51**´** 33. 61**″E**  5203 ft | **SB60 SB62** | 5.448 |
|  |  |  | 34^0^ 09**´** 59. 53**″N**  74^0^ 51**´** 53. 33**″E**  5216 ft |  | 6.048 |
|  |  | 1. Batapora | 34^0^ 10**´** 12. 35**″N**  74^0^ 50**´** 41. 55**″E**  5226ft | **SB37 SB57** | 4.258 |
|  |  |  | 34^0^ 10**´** 06. 83**″N**  74^0^ 50**´** 32. 49**″E**  5224 ft | **SB61** | 4.327 |
|  |  |  | 34^0^ 10**´** 09. 17**″N**  74^0^ 50**´** 29. 65**″E**  5228 ft | **SB45 SB77** | 7.305 |
|  |  | 1. Khunmoah | 34^0^ 04**´** 48. 61**″N**  74^0^ 51**´** 05. 72**″E**  5268 ft |  | 6.201 |
|  |  |  | 34^0^ 04**´** 29. 51**″N**  74^0^ 51**´** 09. 82**″E**  5256 ft | **SB54** | 7.038 |
|  |  |  | 34^0^ 04**´** 52. 30**″N**  74^0^ 51**´** 46. 39**″E**  5255 ft |  | 6.225 |
| **3.** | **Baramulla** | **Block Tujar** | | |  |
|  |  | 1. Tujar | 34^0^ 22**´** 35. 76**″N**  74^0^ 23**´** 46. 10**″E**  5209 ft | **SB31** | 5.305 |
|  |  |  | 34^0^ 22**´** 30. 22**″N**  74^0^ 23**´** 44. 85**″E**  5206ft | **SB6** | 6.003 |
|  |  |  | 34^0^ 22**´** 37. 25**″N**  74^0^ 23**´** 37. 91**″E**  5203 ft | **SB67 SB68** | 5.583 |
|  |  | 1. Bomai | 34^0^ 21**´** 59. 97**″N**  74^0^ 23**´** 12. 91**″E**  5209 ft | **SB49** | 6.110 |
|  |  |  | 34^0^ 22**´** 05. 33**″N**  74^0^ 23**´** 00. 87**″E**  5197 ft | **SB70** | 6.281 |
|  |  |  | 34^0^ 21**´** 58. 72**″N**  74^0^ 23**´** 15. 33**″E**  5198 ft | **SB55** | 7.001 |
|  |  | 1. Brath | 34^0^ 21**´** 39. 22**″N**  74^0^ 25**´** 39. 19**″E**  5204 ft | **SB46 SB47** | 6.342 |
|  |  |  | 34^0^ 21**´** 44. 29**″N**  74^0^ 25**´** 35. 50**″E**  5208 ft |  | 6.391 |
|  |  |  | 34^0^ 21**´** 35. 36**″N**  74^0^ 25**´** 12. 84**″E**  5211 ft | **SB56** | 5.025 |
|  |  | **Block Baramulla** | | |  |
|  |  | 1. Juhama | 34^0^ 14**´** 39. 04**″N**  74^0^ 23**´** 33. 52**″E**  5189 ft | **SB72 SB74** | 7.323 |
|  |  |  | 34^0^ 14**´** 14. 89**″N**  74^0^ 23**´** 55. 59**″E**  5191 ft |  | 7.328 |
|  |  |  | 34^0^ 14**´** 35. 56**″N**  74^0^ 23**´** 21. 71**″E**  5184 ft |  | 8.004 |
|  |  | 1. Kanispora | 34^0^ 12**´** 47. 08**″N**  74^0^ 23**´** 17. 80**″E**  5336 ft | **SB80** | 6.342 |
|  |  |  | 34^0^ 12**´** 44. 95**″N**  74^0^ 23**´** 35. 31**″E**  5360ft | **SB59 SB65** | 6.093 |
|  |  |  | 34^0^ 12**´** 53. 83**″N**  74^0^ 23**´** 40. 14**″E**  5247 ft | **SB79** | 7.776 |
|  |  | 1. Fateh Pora | 34^0^ 12**´** 30. 10**″N**  74^0^ 23**´** 07. 41**″E**  5265 ft | **SB28** | 6.992 |
|  |  |  | 34^0^ 12**´** 33. 79**″N**  74^0^ 22**´** 57. 99**″E**  5376 ft | **SB81** | 6.329 |
|  |  |  | 34^0^ 12**´** 21. 38**″N**  74^0^ 22**´** 31. 76**″E**  5374 ft | **SB75 SB78** | 7.320 |

**Table (S2): Bacterial strains associated with the endosymbiosiome of *Brassica rapa* L. based on their 16s rDNA sequences deposited in NCBI, USA.**

| **Isolate** | **NCBI**  **Accession Number** | **Strain** | **Gene size amplified (bp)** |
| --- | --- | --- | --- |
| SB1 | KY524486 | *Bacillus subtilis* strain skuast1 | 1487 |
| SB2 | KY548645 | *Bacillus foraminis* strain skuast2 | 1484 |
| SB3 | KY612285 | *Enterobacter cloacae* strain SKUAST3 | 1377 |
| SB4 | KY612281 | *Enterobacter cloacae* strain SKUAST-K4 | 1471 |
| SB5 | KY612282 | *Micrococcus endophyticus* strain SKUAST-K5 | 1445 |
| SB6 | KY611893 | *Paenibacillus terrae* strain SKUAST-K6 | 1534 |
| SB7 | KY612284 | *Salinivibrio costicola* strain SKUAST-K7 | 1505 |
| SB8 | KY612272 | *Klebsiella pneumoniae* strain SKUAST-K8 | 1494 |
| SB9 | KY611900 | *Okibacterium endophyticum* strain SKUAST-K9 | 1553 |
| SB10 | KY612277 | *Bacillus firmus* strain SKUAST-K10 | 1502 |
| SB11 | KY612278 | *Salinivibrio costicola* strain SKUAST-K11 | 1542 |
| SB12 | KY612268 | *Pantoea agglomerans* strain SKUAST-K12 | 1427 |
| SB13 | KU674947 | *Bacillus subtilis* strain smppsap1 | 1527 |
| SB14 | KU883268 | *Bacillus subtilis* strain smppsap2 | 1523 |
| SB15 | KY612269 | *Pseudomonas chlororaphis* strain SKUAST-K15 | 1361 |
| SB16 | KY612283 | *Pseudomonas koreensis* strain SKUAST-K16 | 1461 |
| SB17 | KY612287 | *Micrococcus endophyticus strain SKUAST-K17* | 1482 |
| SB18 | KY612276 | *Pseudomonas mendocina* strain SKUAST-K18 | 1541 |
| SB19 | KY612273 | *Arthrobacter endophyticus* strain SKUAST-K19 | 1573 |
| SB20 | KY612270 | *Pseudomonas fuscovaginae* strain SKUAST-K20 | 1431 |
| SB21 | KY612271 | *Pseudomonas brassicacearum* strain SKUAST-K21 | 1467 |
| SB22 | KY612280 | *Microbacterium ulmi* strain SKUAST-K22 | 1480 |
| SB23 | KY612279 | *Microbacterium phyllosphaerae* strain SKUAST-K23 | 1547 |
| SB24 | KY612275 | *Serratia marcescens* strain SKUAST-K24 | 1565 |
| SB25 | KY612288 | *Pseudomonas brassicacearum* strain SKUAST-K25 | 1539 |
| SB26 | KY612289 | *Pseudomonas rhizosphaerae* strain SKUAST-K26 | 1558 |
| SB27 | KY612291 | *Pseudomonas cedrin*a strain SKUAST-K27 | 1531 |
| SB28 | KU883269 | *Pseudomonas lutea* strain smppsap3 | 1508 |
| SB29 | KY612292 | *Bacillus flexus* strain SKUAST-K29 | 1520 |
| SB30 | KY612290 | *Bacillus herbersteinensis* strain SKUAST-K30 | 1488 |
| SB31 | KY621796 | *Comamonas terrigena* strain SKUAST-K31 | 1482 |
| SB32 | KY621553 | *Pseudomonas fragi* strain SKUAST-K32 | 1504 |
| SB33 | KY621803 | *Pseudomonas putida* strain SKUAST-K33 | 1522 |
| SB34 | KY621798 | *Curvibacter gracilis* strain SKUAST-K34 | 1532 |
| SB35 | KY621551 | *Delftia acidovorans* strain SKUAST-K35 | 1533 |
| SB36 | KY628826 | *Pseudomonas brenneri* strain SKUAST-K36 | 1536 |
| SB37 | KY621804 | *Pseudomonas fluorescens* strain SKUAST-K37 | 1505 |
| SB38 | KY621552 | *Pseudomonas marginalis* strain SKUAST-K38 | 1493 |
| SB39 | KY621801 | *Pseudomonas marginalis* strain SKUAST-K39 | 1475 |
| SB40 | KY621797 | *Pseudomonas chlororaphis* strain SKUAST-K40 | 1392 |
| SB41 | KY646012 | *Pseudomonas orientalis* strain SKUAST-K41 | 1494 |
| SB42 | KY646002 | *Pseudomonas azotoformans* strain SKUAST-K42 | 1478 |
| SB43 | KY646015 | *Bacillus amyloliquefaciens* strain SKUAST-K43 | 1481 |
| SB44 | [KY612274](https://www.ncbi.nlm.nih.gov/sites/entrez?cmd=Search&db=nucleotide&term=KY612274.1&dopt=GenBank) | *Paenibacillus polymyxa* strain SKUAST-K6 | 1534 |
| SB45 | KY646007 | *Bacillus subtilis* strain SKUAST-K45 | 1495 |
| SB46 | KY646014 | *Bacillus velezensis* strain SKUAST-K46 | 1483 |
| SB47 | KY646005 | *Pseudomonas mucidolens* strain SKUAST-K47 | 1529 |
| SB48 | KY646009 | *Bacillus* sp. strain SKUAST-K48 | 1515 |
| SB49 | KY646001 | *Bacillus velezensis* strain SKUAST-K49 | 1573 |
| SB50 | KY646008 | *Pseudomonas synxantha* strain SKUAST-K50 | 1589 |
| SB51 | KU883270 | *Pseudomonas fluorescens* strain smppsap4 | 1502 |
| SB52 | KY646011 | *Bacillus licheniformis* strain SKUAST-K52 | 1514 |
| SB53 | KY646003 | *Pseudomonas mucidolens* strain SKUAST-K53 | 1475 |
| SB54 | KY646013 | *Pseudomonas lurida* strain SKUAST-K54 | 1480 |
| SB55 | KY646004 | *Pseudomonas fluorescens* strain SKUAST-K55 | 1471 |
| SB56 | KY646006 | *Pseudomonas grimontii* strain SKUAST-K56 | 1470 |
| SB57 | KY646017 | *Pseudomonas panacis* strain SKUAST-K57 | 1474 |
| SB58 | KY646010 | *Pseudomonas chlororaphis* strain SKUAST-K58 | 1474 |
| SB59 | KY646021 | *Bacillus pumilus* strain SKUAST-K59 | 1525 |
| SB60 | KY646030 | *Pseudomonas rhodesiae* strain SKUAST-K60 | 1471 |
| SB61 | KY646027 | *Micrococcus luteus* strain SKUAST-K61 | 1512 |
| SB62 | KY646018 | *Micrococcus* sp. strain SKUAST-K62 | 1463 |
| SB63 | KY646026 | *Pseudomonas veronii* strain SKUAST-K63 | 1476 |
| SB64 | KU883601 | *Bacillus pumilus* strain smppsap6 | 1526 |
| SB65 | KY646031 | *Bacillus amyloliquefaciens* strain SKUAST-K65 | 1519 |
| SB66 | KY646023 | *Pseudomonas lurida* strain SKUAST-K66 | 1546 |
| SB67 | KY646028 | *Pseudomonas veronii* strain SKUAST-K67 | 1520 |
| SB68 | KY646019 | *Pseudomonas poae* strain SKUAST-K68 | 1529 |
| SB69 | KY646020 | *Bacillus amyloliquefaciens* strain SKUAST-K69 | 1539 |
| SB70 | KY646029 | *Pseudomonas* sp. strain SKUAST-K70 | 1507 |
| SB71 | KY646016 | *Bacillus licheniformis* strain SKUAST-K71 | 1557 |
| SB72 | KY646025 | *Pseudomonas veronii* strain SKUAST-K72 | 1488 |
| SB73 | KY646024 | *Pseudomonas* sp. strain SKUAST-K73 | 1591 |
| SB74 | KY646039 | *Pseudomonas palleroniana* strain SKUAST-K74 | 1437 |
| SB75 | KY646036 | *Bacillus polyfermenticus* strain SKUAST-K75 | 1571 |
| SB76 | KY646041 | *Pseudomonas fluorescens* strain SKUAST-K76 | 1520 |
| SB77 | KY646034 | *Pseudomonas veronii* strain SKUAST-K77 | 1483 |
| SB78 | KY646035 | *Pseudomonas* sp. strain SKUAST-K78 | 1426 |
| SB79 | KY646037 | *Bacillus amyloliquefaciens* strain SKUAST-K79 | 1515 |
| SB80 | KY646038 | *Bacillus* sp. strain SKUAST-K80 | 1519 |
| SB81 | KY646040 | *Pseudomonas palleroniana* strain SKUAST-K81 | 1445 |
